# Microwave irradiation: synthesis and characterization of α-ketoamide and bis (α-ketoamide) derivatives via the ring opening of *N*-acetylisatin

**DOI:** 10.1186/1752-153X-8-27

**Published:** 2014-04-28

**Authors:** Ayman El-Faham, Sherine N Khattab, Hazem A Ghabbour, Hoong-Kun Fun, M Rafiq H Siddiqui

**Affiliations:** 1 Department of Chemistry, College of Science, King Saud University, P.O. Box 2455, 11451 Riyadh, (Kingdom of Saudi Arabia; 2 Department of Chemistry, Faculty of Science, Alexandria University, P.O. Box 426, Ibrahimia 21321, Alexandria, Egypt; 3 Department of Pharmaceutical Chemistry, College of Pharmacy, King Saud University, P.O. Box. 2457, 11451 Riyadh, Saudi (Kingdom of Arabia

**Keywords:** *N*-acetylisatin, *N*-propionylisatin, Microwave irradiation, α-ketoamide, *bis*- α-ketoamide, X-ray crystallography

## Abstract

**Background:**

The carbonyl group at position 2 of *N*-acetylisatin behaves as an amide which is more susceptible to nucleophilic attack via ring-opening in the presence of nucleophiles. Because of this behavior, in the present work we describe the microwave synthesis of a series of α-ketoamide and *bis*-(α-ketoamide) derivatives via the facile ring-opening of *N*-acylisatin with different amines and diamines. The microwave irradiation afforded the product in less reaction time, higher yield and purity. Reaction of *N*-acylisatin with methanol under microwave irradiation afforded the α-phenylglyoxyl methyl ester derivatives with excellent yields and purities. Aminolysis of the ester derivatives with piperidine and morpholine afforded the same α-ketoamide derivatives obtained from direct aminolysis of *N*-acylisatin. The structures of the synthesized compounds were confirmed by FT-IR, NMR, X-ray and elemental analysis.

**Results:**

Reaction of *N*-acetylisatin and *N*-propoionylsatin with different amines and diamines afforded a series of α-ketoamide and *bis*-(α-ketoamide) derivatives respectively via the ring opening of *N*-acylisatins. The reaction was performed under conventional condition as well as microwave irradiation. The microwave irradiation afforded the product in less reaction time, higher yield and purity. Reaction of *N*-acylisatin with methanol under microwave irradiation afforded the α-phenylglyoxyl methyl ester derivatives in excellent yields and purities as observed from their spectral data. A plausible mechanism involves nucleophilic attack by methanol at C2 carbonyl carbon of *N*-acetylisatin and subsequent ring opening to generate the α-ketoester. Aminolysis of α-ketoester with amine afforded the same α-ketoamide which is obtained by direct aminolysis of *N*-acylisatin. The IR, NMR spectra, microanalyses, and single crystal X-ray diffraction confirmed the structures of the synthesized compounds.

**Conclusions:**

In conclusion, we have demonstrated that microwave irradiation could be employed efficiently for the synthesis of biologically important α-ketoamide and *bis*-(α-ketoamide) derivatives. The microwave irradiation has more advantageous over the classical method with regard to reaction time, solvent quantity, and product yield. Reaction of *N*-acylisatin with methanol under microwave irradiation afforded the α-phenylglyoxyl methyl ester derivatives with excellent yields and purities. Aminolysis of the methyl ester derivatives with amine under microwave irradiation afford the same α-ketoamide derivatives as obtained from direct aminolysis of *N*-acylisatins.

## Background

Microwave irradiation (MW) has emerged as a powerful technique offering simple, clean, fast, efficient, and economical method for the synthesis of a large number of biologically active molecules [1-6]. The application of microwave irradiation in organic synthesis has been the focus of considerable attention in recent years and is becoming an increasingly popular technology [7-14].

The carbonyl group at position 2 of *N*-acetylisatin behaves as an amide carbonyl group which is opposed to the conventional amide carbonyl functionality in isatin [15]. Because of this behavior, *N*-acetylisatin is more susceptible to nucleophilic attack via ring-opening in the presence of nucleophiles, such as amines and alcohols. This ring-opening reaction led to numerous synthetic methods for preparation of α-ketoamides [16-22], which are of interest in organic and medicinal chemistry [23-27].

In continuation of our earlier work [11,12], we present herein the reaction of *N*-acetyl- and *N*-propionylisatin in methanol with different amines and diamines under microwave irradiation to afford α-ketoester, series of α-ketoamide and *bis*-(α-ketoamide) derivatives in excellent yields and purities.

## Results and discussion

**Scheme 1:**
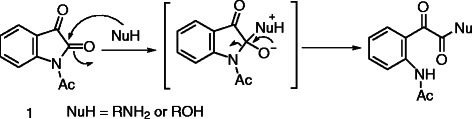
**General mechanism for the reaction of *N*-acylisatin with amine or alcohol.**

Isatin and its derivatives undergo nucleophilic attack at the carbonyl group at position 2 and/or 3. The chemoselectivity of these reactions depends on the nature of the substituent attached to the isatin nucleus, on the nature of the nucleophiles, and the nature of the group attached to the nitrogen atom of the isatin nucleus [15]. *N-*acetylisatin (**1**) undergoes ring-opening reaction with primary amines and alcohols as reported in the literature (Scheme 1) [28].

**Scheme 2:**
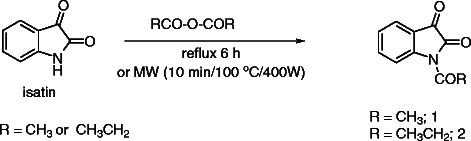
**Synthesis of *N*-acylisatin.**

*N*-Acetylisatin (**1**) and *N*-propionylisatin (**2**) were initially prepared by reaction of isatin with acetic anhydride or propanoic anhydride respectively (Scheme 2), using the same reported conventional conditions [29]. Compound **1** and **2** were also prepared by microwave irradiation for 10 min at 100°C/400 W using a multimode reactor (Synthos 3000 Aton Paar, GmbH, 1400 W maximum magnetron) (Scheme 2). The microwave irradiation afforded the product in less reaction time, higher yield and purity than conventional heating.

**Scheme 3:**
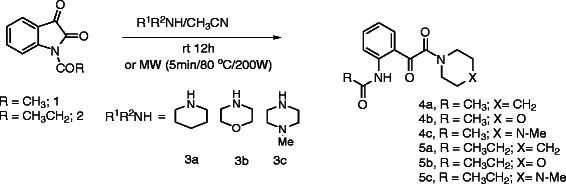
**Reaction of *N*-acylisatin with secondary amines.**

*N*-Acetylisatin, **1** and *N*-propionylisatin, **2** were reacted with different secondary amines **3a**-**c** at room temperature in acetonitrile as a solvent to afford α-ketoamide **4a**-**c** and **5a**-**c** in yield 64-72% (Scheme 3). The microwave irradiation of **1** and **2** with different amines **3a**-**c** afforded the final products **4a**-**c** and **5a**-**c** in higher yields and purities than conventional method (Experimental section). The structures of the prepared compounds were confirmed by IR, NMR (^1^H-NMR and ^13^C-NMR) and elemental analysis.

**Figure 1:**
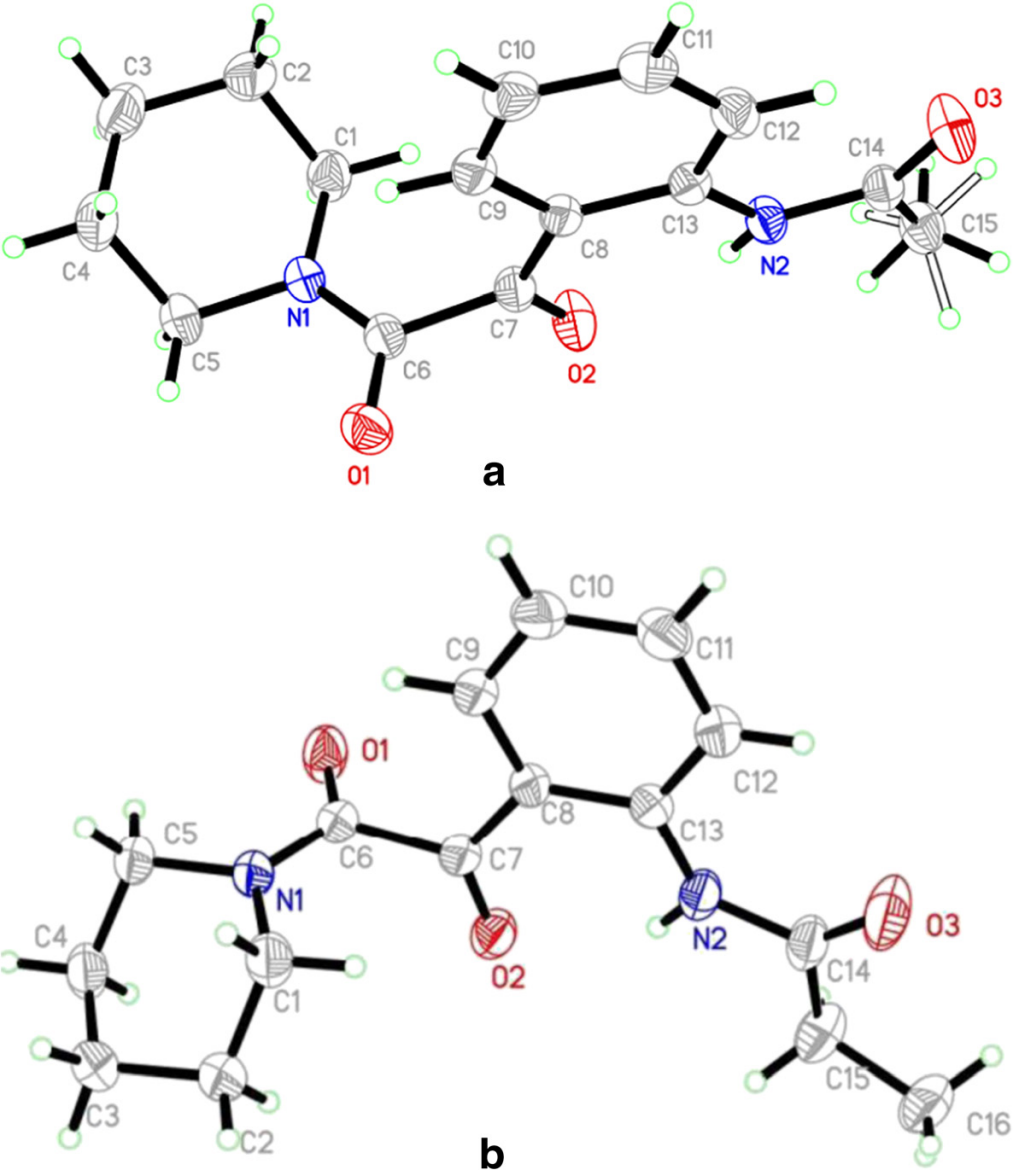
**ORTEP representation of the crystal structure of 4a and 5a. (a)** Ortep of N-(2-(2-oxo-2-(piperidin-1-yl)acetyl)phenyl)acetamide 4a CCDC 943007. **(b)** Ortep of N-(2-(2-oxo-2-(piperidin-1-yl)acetyl)phenyl)propionamide **5a** CCDC 945756.

As prototype the IR spectrum of **4a** showed four characteristic peaks at 3399, 1691, 1630, 1605 cm^−1^, corresponding to the NH, α-ketoamide (*CO*CONH) and two CONH, respectively. The ^1^H-NMR of **4a** agreed well with the structure, showing resonance peaks located at δ 1.55 (m, 2H, CH_2_-*CH*_*2*_-CH_2,_ piperidine moiety), 1.70 (m, 4H, C*H*_*2*_-CH_2_-C*H*_*2*,_ piperidine moiety), 2.25 (s, 3H, COC*H*_*3*_), 3.30 and 3.69 (two m, 4H, C*H*_*2*_-N-C*H*_*2*,_ piperidine moiety), 7.12 (t, 1H, ArH), 7.65 (m, 2H, ArH), 8.88 (d, 1H, ArH), 11.30 (s, 1H, NH, D_2_O exchangeable). The ^13^C-NMR of **4a** also confirmed the structure, showing the characteristic signals at δ 164.5, 169.6 and 196.3 ppm, related to the two carbonyl of the amide group and one related to the α-ketoamide group respectively along with the rest of the expected carbon signals of the compound. X-Ray single crystal structure determination of compound **4a** and **5a** also confirmed the structure of the products (Figure 1a and b). Crystal data and structure refinement details for compound **4a** and **5a** are shown in Table 1.

**Table 1:** Crystal data and structure refinement details for compounds 4a and 5a

	**4a**	**5a**
**Molecular formula**	C_15_H_18_N_2_O_3_	C_16_H_20_N_2_O_3_
**Formula weight**	274.31	288.34
**Crystal system**	Orthorhombic	Triclinic
**Space group**	*P*2_1_2_1_2_1_	*P1*
**Unit cell dimensions**	a = 10.0002 (2) Å	a = 5.1016 (2) Å, α = 83.188 (2)°
	b = 11.7485 (2) Å	b = 8.8474 (3) Å, β = 81.358 (3)°
	c = 11.9183 (2) Å	c = 16.9672 (5) Å, γ = 83.745 (3)°
**Volume**	1400.25 (4) Å^3^	748.54 (4) Å^3^
**Z, Calculated density**	1.301 g cm^−3^	1.279 g cm^−3^
**F(000)**	584	308
**Crystal size**	0.82 × 0.67 × 0.43 mm	0.43 × 0.25 × 0.23 mm
**θ range for data collection**	θ_max_ = 71.8°, θ_min_ = 5.3°	θ_max_ = 65.0°, θ_min_ = 2.7°
**Limiting indices**	−12 < =h < =12, −12 < =k < =14, −13 < =l < =14	−4 < =h < =5, −10 < =k < =10, −19 < =l < =19
**Reflections collected/unique**	9719/2682 [*R*_int_ = 0.021]	8120/ 2453 [*R*_int_ = 0.111]
**Completeness**	to theta 71.6 = 98.8%	to theta 65.0 = 96.8%
**Absorption correction**	multi-scan SADABS Bruker 2009	multi-scan SADABS Bruker 2009
**Refinement method**	Full-matrix least-squares on *F*^*2*^	Full-matrix least-squares on *F*^*2*^
**Goodness-of-fit on *F***^***2***^	1.03	1.64
**CCDC number**	943007	945756

**Scheme 4:**
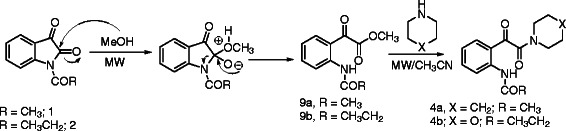
**Reaction mechanism of *N*-acylisatin with methanol.**

Alternatively, the *N*-acylisatin **1** and **2** were dissolved in dry methanol and then irradiated under microwave irradiation for 3–5 min followed by cooling to afford the products **9a** and **9b** in yield 95% and 91%, respectively (Scheme 4). A plausible mechanism involves nucleophilic attack by methanol at C2 carbonyl carbon of *N*-acetylisatin and subsequent ring opening to generate the α-keto ester (Scheme 4).

The IR spectrum of **9a** showed three carbonyl groups at 1747 (CO-ester), 1696 (α-CO), and 1657 (CONH) cm^−1^. The ^1^HNMR spectrum for **9a** showed singlet peaks at δ 2.25 and 3.99 ppm related to (NHCOC*H*_*3*_) and (COOC*H*_*3*_) respectively. The ^13^CNMR of **9a** showed a characteristic peaks for *CO*-ester and *α-CO*- at δ 169.6 and 190.3 ppm respectively.

**Figure 2:**
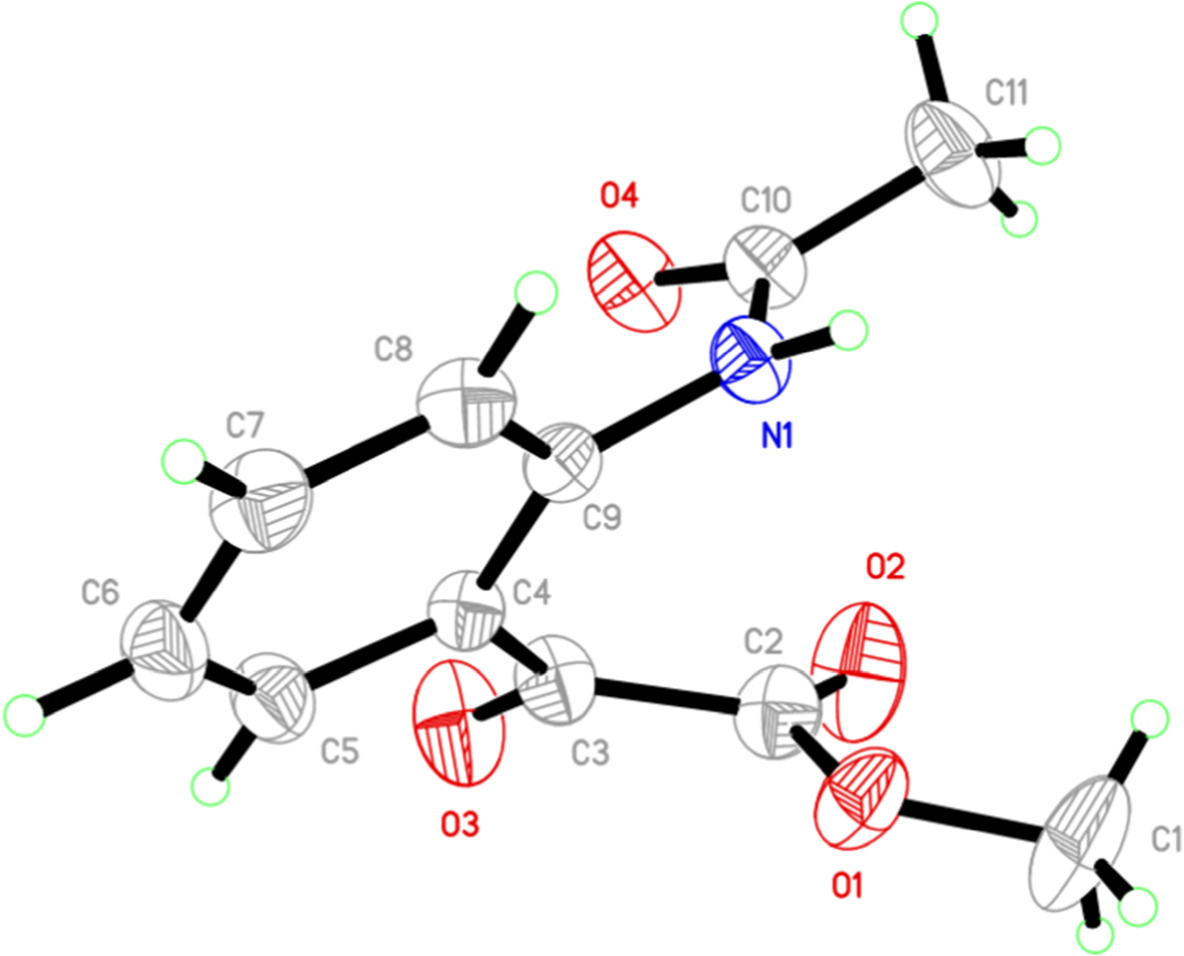
Ortep of methyl 2-(2-acetamidophenyl)-2-oxoacetate 9a CCDC No. 942706.

The X-ray crystallographic structure for **9a** (Figure 2, Table 2) also confirmed its structure. Crystal data for compound **9a** are shown in Table 2. In the crystal, the molecules are linked via intermolecular N—H----O and C—H-----O hydrogen bonds.

**Table 2:** Crystal data and structure refinement details for compound 9a

	**9a**
**Molecular formula**	C_11_H_11_NO_4_
**Formula weight**	221.21
**Crystal system**	Monoclinic
**Space group**	*P*2_1_/*c*
**Unit cell dimensions**	a = 7.3649 (2)Å
	b = 18.6848 (5)Å
	c = 9.3738 (3)Å, β = 121.126 (2)°
**Volume**	1104.23 (5) Å^3^
**Z, Calculated density**	1.331 g cm^−3^
**F(000)**	464
**Crystal size**	0.52 × 0.48 × 0.22 mm
**θ range for data collection**	θ_max_ = 70.1°, θ_min_ = 4.7°
**Limiting indices**	−8 < =h < =7, −22 < =k < =22, −9 < =l < =11
**Reflections collected/unique**	7581/2018 [*R*_int_ = 0.022]
**Completeness**	to theta 70.1 = 96.2%
**Absorption correction**	multi-scan, SADABS Bruker 2009
**Refinement method**	Full-matrix least-squares on *F*^*2*^
**Goodness-of-fit on *F***^***2***^	1.11
**CCDC number**	942706

Reaction of α-phenylglycoxyl methyl ester derivatives **9a** and **9b** with piperidine and morpholine under the conventional heating (2 h) and microwave irradiation (2–5 min/80°C/200 W) afforded the same product **4a-b** and **5a-b**, respectively as obtained from the direct aminolysis of *N*-acylisatin **1** and **2** (Scheme 4).

**Scheme 5:**
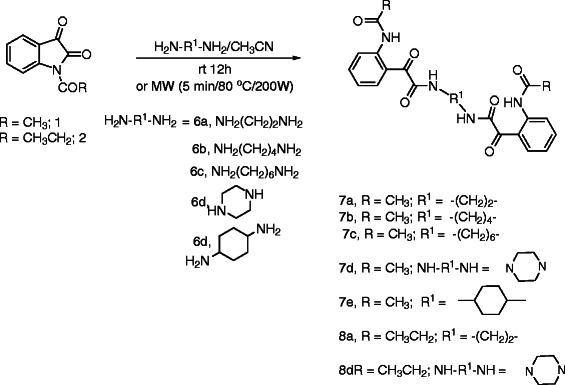
**Reaction of *N*-acylisatin with diamines.**

Reaction of *N*-acetylisatin, **1** and *N*-propionylisatin, **2** with different diamines **6a-e** in ratio (2:1) at room temperature for 12 h in acetonitrile as solvent afforded *bis*-(α-ketoamide) **7a-e 8a** and **8e** in yields of 68-73% (Scheme 5). The microwave irradiation of **1** and **2** with diamines afforded the products **7a-e**, **8a** and **8e** in higher yield and purity (Experimental section). The structures of the prepared compounds were confirmed by IR, NMR (^1^H-NMR and ^13^C-NMR) and elemental analysis

## Experimental section

### Materials and methods

The solvents used were of HPLC reagent grade. Melting points were determined with a Mel-Temp apparatus. Fourier transform infrared spectroscopy (FT-IR) spectra were recorded on Nicolet 560. Nuclear magnetic resonance spectra (^1^H NMR and ^13^C NMR spectra) were recorded on a JOEL 400 MHz spectrometer with chemical shift values reported in δ units (ppm) relative to an internal standard. X-Ray data collection was carried out on Bruker SMART APEX II CCD diffractometer, cell refinement: SAINT; data reduction: SAINT; program used to solve structure: SHELXS; program used to refine structure: SHELXL; molecular graphics: SHELXTL; software used to prepare material for publication: SHELXTL [30] and PLATON [31]. The microwave irradiation employed a multimode reactor (Synthos 3000, Aton Paar GmbH, and 1400 W maximum magnetron). Elemental analyses were performed on Perkin-Elmer 2400 elemental analyzer, and the values found were within ±0.3% of the theoretical values. Follow-up of the reactions and checks of the purity of the compounds was done by TLC on silica gel-protected aluminum sheets (Type 60 GF254, Merck). The compounds were named using Chem. Draw Ultra version 11, Cambridge soft Corporation.

### General method for synthesis of α-ketoamide and *bis*-(α-ketoamide) derivatives

#### Method A (Conventional Procedure)

To a solution of *N*-acetylisatin **1** or *N*-propionylisatin **2** (10 mmol) in acetonitrile (20 mL), secondary amine **3a-c** or diamine **6a-e** in ratio (1:1 and 2:1, respectively) was added at room temperature. The reaction mixture was stirred at room temperature for 12 h. On the next day, the solvent was removed under vacuum and the crude product was recrystallized from dichloromethane and hexane (1:2). In the case of diamine, the precipitate was filtered, washed with acetonitrile (5 mL), and dried under vacuum to afford the pure product.

#### Method B (Microwave-Irradiation)

The reaction was performed using a multimode reactor (Synthos 3000 Aton Paar, GmbH, 1400 W maximum magnetron). The initial step was conducted with 4-Teflon vessels rotor (MF 100) that allows the reactions to be processed 4 at a time under the same conditions. In each vessel *N*-acylisatin mixed with different amine or diamine in small amount of acetonitrile (2–5 mL). The individual vessels were purged with nitrogen gas for 2 min and then placed in the corresponding rotor. The vessels were heated for 3 min at 80°C and held at the same temperature for a further 2 min at 200 W. Cooling was accomplished by a fan (5 min); the final product was precipitated after cooling, filtered, dried under vacuum, and then recrystallized from dichloromethane-hexane (1:2).

### *N*-[2-(2-Oxo-2-(piperidin-1-yl)acetyl)phenyl]acetamide 4a (^1^H NMR and ^13^C NMR attached as a supporting information; Additional file 1)

Light brown crystals; mp 132-133°C; yield (65% method A, Lit. [17] mp 130-132 °C); (91% method B); IR (KBr, cm^−1^): 3399 (NH), 1691 (α-*CO*CON), 1630 (CO-amide), 1605 (CO-amide). ^1^H-NMR (CDCl_3_): δ = 1.55 (m, 2H, CH_2_), 1.70 (m, 4H, 2CH_2_), 2.25 (s, 3H, COCH_3_), 3.30 (m, 2H, CH_2_), 3.69 (m, 2H, CH_2_), 7.12 (t, *J =* 8.08 Hz, 1H, ArH), 7.65 (m, 2H, ArH), 8.88 (d, *J =* 8.80 Hz, 1H, ArH), 11.30 (s, 1H, NH, D_2_O exchangeable). ^13^C-NMR (CDCl_3_): δ = 24.3, 25.5, 25.7, 26.2, 42.3, 47.2, 117.9, 120.7, 122.8, 133.6, 136.9, 142.5, 164.5 (CO-amide), 169.6 (CO-amide), 196.2 (α-*CO*CON). Anal. Calcd for C_15_H_18_N_2_O_3_: C, 65.68; H, 6.61; N, 10.21. Found: C, 65.56; H, 6.67; N, 10.33.

### *N*-[2-(2-Morpholino-2-oxoacetyl)phenyl]acetamide 4b (^1^H NMR and ^13^C NMR attached as a supporting information; Additional file 1)

Light brown crystals; mp 111-113°C; yield (69% method A); (93% method B); IR (KBr, cm^−1^): 3399 (NH), 1693 (α-*CO*-CON), 1635 (CO-amide), 1608 (CO-amide). ^1^H-NMR (CDCl_3_): δ = 2.24 (s, 3H, COCH_3_), 3.34 (t, *J =* 5.12 Hz, 2H, CH_2_), 3.65 (t, *J =* 4.40 Hz, 2H, CH_2_), 3.77 (m, 4H, 2CH_2_), 7.13 (t, *J =* 7.36 Hz, 1H, ArH), 7.67 (m, 2H, ArH), 8.78 (d, *J =* 8.04 Hz, 1H, ArH), 11.21 (s, 1H, NH, D_2_O exchangeable) ppm; ^13^C-NMR (CDCl_3_): δ = 25.7, 41.3, 46.4, 66.7, 117.8, 120.8, 122.9, 133.53, 137.2, 142.6, 164.6 (CO-amide), 169.6 (CO-amide), 195.4 (α-*CO*CON) ppm. Anal. Calcd for C_14_H_16_N_2_O_4_: C, 60.86; H, 5.84; N, 10.14. Found: C, 60.78; H, 5.77; N, 10.31.

### *N*-[2-(2-(4-Methylpiperazin-1-yl)-2-oxoacetyl)phenyl]acetamide 4c (^1^H NMR and ^13^C NMR attached as a supporting information; Additional file 1)

Light brown crystals; mp 113-115°C; yield (64% method A); (89% method B); IR (KBr, cm^−1^): 3396 (NH), 1692 (α-*CO*-CON), 1633 (CO-amide), 1608 (CO-amide). ^1^H-NMR (CDCl_3_): δ 2.23 (s, 3H, COCH_3_), 2.30 (s, 3H, N-CH_3_), 2.35 (t, *J =* 5.01 Hz, 2H, CH_2_), 2.49 (t, *J =* 5.12 Hz, 2H, CH_2_), 3.33 (t, *J =* 5.12 Hz, 2H, CH_2_), 3.77 (t, *J =* 5.12 Hz, 2H, CH_2_), 7.12 (t, *J =* 7.32 Hz, 1H, ArH), 7.63 (m, 2H, ArH), 8.77 (d, *J =* 8.80 Hz, 1H, ArH), 11.24 (s, 1H, NH, D_2_O exchangeable) ppm; ^13^C-NMR (CDCl_3_): δ = 25.6, 41.3, 46.0, 46.1, 54.5, 54.9, 117.9, 120.7, 122.8, 133.6, 137.1, 142.5, 164.4 (CO-amide), 169.8 (CO-amide), 195.7 (α-*CO*CON). Anal. Calcd for C_15_H_19_N_3_O_3_: C, 62.27; H, 6.62; N, 14.52. Found: C, 62.38; H, 6.71; N, 14.69.

### *N*-[2-(2-Oxo-2-(piperidin-1-yl)acetyl)phenyl]propionamide 5a (^1^H NMR and ^13^C NMR attached as a supporting information; Additional file 2)

Light brown crystals; mp 102-103°C; yield (70% method A); (95% method B); IR (KBr, cm^−1^): 3396 (NH), 1695 (α-*CO*-CON), 1630 (CO-amide), 1608 (CO-amide). ^1^H-NMR (CDCl_3_): δ = 1.26 (t, *J =* 7.32 Hz, 3H, CH_2_CH_3_), 1.53 (m, 2H, CH_2_), 1.68 (m, 4H, 2CH_2_), 2.49 (q, *J =* 7.36 Hz, 2H, CH_2_CH_3_), 3.26 (t, *J =* 5.12 Hz, 2H, CH_2_), 3.68 (m, 2H, CH_2_), 7.12 (t, *J =* 8.08 Hz, 1H, ArH), 7.64 (m, 2H, ArH), 8.81 (d, *J =* 8.80 Hz, 1H, ArH), 11.31 (s, 1H, NH, D_2_O exchangeable) ppm; ^13^C-NMR (CDCl_3_): δ = 9.5, 24.4, 25.4, 25.7, 26.2, 31.8, 42.3, 47.2, 120.7, 118.0, 122.7, 133.5, 136.9, 142.6, 164.5 (CO-amide), 173.4 (CO-amide), 196.2 (α-*CO*CON) ppm. Anal. Calcd for C_16_H_20_N_2_O_3_: C, 66.65; H, 6.99; N, 9.72. Found: C, 66.38; H, 6.81; N, 9.98.

### *N*-[2-(2-Morpholino-2-oxoacetyl)phenyl]propionamide 5b (^1^H NMR and ^13^C NMR attached as a supporting information; Additional file 2)

Light brown crystals; mp 112-112°C; yield (69% method A); (93% method B); IR (KBr, cm^−1^): 3394 (NH), 1691 (α-CO), 1632 (CO-amide), 1610 (CO-amide). ^1^H-NMR (CDCl_3_): δ = 1.29 (t, *J =* 7.32 Hz, 3H, CH_2_CH_3_), 2.48 (q, *J =* 7.36Hz, 2H, CH_2_CH_3_), 3.36 (t, *J =* 5.12 Hz, 2H, CH_2_), 3.66 (t, *J =* 5.12 Hz, 2H, CH_2_), 3.78 (brs, 4H, 2CH_2_), 7.13 (t, *J =* 7.13 Hz, 1H, ArH), 7.67 (m, 2H, ArH), 8.83 (d, *J =* 8.80 Hz, 1H, ArH), 11.24 (s, 1H, NH, D_2_O exchangeable) ppm; ^13^C-NMR (CDCl_3_): δ = 9.5, 31.8, 41.7, 46.4, 66.7, 117.8, 120.9, 122.7, 133.5, 137.2, 142.8, 164.6 (CO-amide), 173.4 (CO-amide), 195.4 (α-*CO*CON) ppm. Anal. Calcd for C_15_H_18_N_2_O_4_: C, 62.06; H, 6.25; N, 9.65. Found: C, 62.18; H, 6.17; N, 9.89.

### *N*-[2-(2-(4-Methylpiperazin-1-yl)-2-oxoacetyl)phenyl]propionamide 5c (^1^H NMR and ^13^C NMR attached as a supporting information; Additional file 2)

Light brown crystals; mp 90-92°C; yield (72% method A); (89% method B); IR (KBr, cm^−1^): 3366 (NH), 1710 (α-CO), 1644 (CO-amide), 1581 (CO-amide). ^1^H-NMR (CDCl_3_): δ = 1.26 (s, 3H, CH_2_CH_3_), 2.30 (s, 3H, N-CH_3_), 2.35 (t, *J =* 5.12 Hz, 2H, CH_2_), 2.51 (m, 4H, CH_2_CH_3_, CH_2_), 3.35 (t, *J =* 4.80 Hz, 2H, CH_2_), 3.77 (t, *J =* 5.16 Hz, 2H, CH_2_), 7.12 (t, *J =* 8.08 Hz, 1H, ArH), 7.63 (m, 2H, ArH), 8.81 (d, *J =* 8.80 Hz, 1H, ArH), 11.27 (s, 1H, NH, D_2_O exchangeable) ppm; ^13^C-NMR (CDCl_3_): δ = 9.5, 31.8, 41.3, 46.0, 46.4, 54.5, 54.9, 117.9, 120.8, 122.7, 133.6, 137.1, 142.7, 164.5 (CO-amide), 173.4 (CO-amide), 195.7 (α-*CO*CON) ppm. Anal. Calcd for C_16_H_21_N_3_O_3_: C, 63.35; H, 6.98; N, 13.85; Found: C, 63.21; H, 6.87; N, 14.03.

### *N, N'*-(Ethane-1,2-diyl)*bis*[2-(2-acetamidophenyl)-2-oxoacetamide] 7a (^1^H NMR and ^13^C NMR attached as a supporting information; Additional file 3)

White solid; mp 220-221°C (dec.); yield (72% method A); (92% method B); IR (KBr, cm^−1^): 3351 (NH), 1685 (α-CO), 1653 (CO-amide), 1587 (CO-amide). ^1^H-NMR (DMSO-d_6_): δ = 2.06 (s, 6H,2 COCH_3_), 3.36 (m, 4H, 2CH_2_), 7.20 (t, *J =* 7.32 Hz, 2H, ArH), 7.65 (t, *J =* 8.08 Hz, 2H, 2 NH), 7.67(d, *J =* 8.08 Hz, 2H, 2ArH), 7.81 (d, *J =* 8.08 Hz, 2H, ArH), 8.84 (d, 2H, ArH), 11.55 (s, 2H, 2NH) ppm; ^13^C-NMR (DMSO-d_6_): δ = 24.5, 39.9, 122.1, 124.0, 124.8, 134.4, 138.8, 164.2 (CO-amide), 169.3 (CO-amide), 191.2 (α-*CO*CON) ppm. Anal. Calcd for C_22_H_22_N_4_O_6_: C, 60.27; H, 5.06; N, 12.78; Found: C, 60.38; H, 5.11; N, 12.69.

### *N, N*'-(Butane-1,4-diyl)*bis*[2-(2-acetamidophenyl)-2-oxoacetamide] 7b (^1^H NMR and ^13^C NMR attached as a supporting information; Additional file 3)

White solid; mp 205-207°C (dec.); yield (70% method A); (90% method B); IR (KBr, cm^−1^): 3258 (NH), 1698 (α-CO), 1663 (CO-amide), 1602 (CO-amide). ^1^H-NMR (DMSO-d_6_): δ 1.55 (m, 4H, 2CH_2_), 2.05 (s, 6H, 2COCH_3_), 3.23 (m, 4H, 2 CH_2_), 7.23 (t, *J =* 8.04 Hz, 2H, ArH), 7.64 (m, 4H, 2NH, 2ArH), 7.89 (d, *J =* 8.08 Hz, 2H, ArH), 8.75 (t, *J =* 5.16 Hz, 2H, ArH), 11.60 (s, 2H, 2NH) ppm; ^13^C-NMR (DMSO-d_6_): δ = 24.6, 26.8, 39.6, 121.9, 124.0, 124.2, 132.3, 134.7, 139.2, 164.3 (CO-amide), 169.4 (CO-amide), 192.4 (α-*CO*CON) ppm. Anal. Calcd for C_24_H_26_N_4_O_6_: C, 61.79; H, 5.62; N, 12.01. Found: C, 61.87; H, 5.71; N, 12.19.

### *N, N'*-(Hexane-1,6-diyl)*bis*[2-(2-acetamidophenyl)-2-oxoacetamide] 7c (^1^H NMR and ^13^C NMR attached as a supporting information; Additional file 3)

White solid; mp 176-178°C (dec.); yield (72% method A); (94% method B); IR (KBr, cm^−1^): 3257 (NH), 1698 (α-CO), 1662 (CO-amide), 1608 (CO-amide). ^1^H-NMR (DMSO-d_6_): δ = 1.33 (m, 4H, 2CH_2_), 1.51 (m, 4H, 2CH_2_), 2.07 (s, 6H, 2 COCH_3_), 3.21 (m, 4H, 2 CH_2_), 7.23 (t, *J =* 7.32 Hz, 2H, ArH), 7.62 (m, 4H, 2NH, 2ArH), 7.91 (d, *J =* 8.08 Hz, 2H, ArH), 8.73 (t, *J =* 5.88 Hz, 2H, ArH), 10.91 (s, 2H, 2NH) ppm; ^13^C-NMR (DMSO-d_6_): δ = 24.7, 26.6, 29.6, 39.1, 121.9, 124.0, 124.1, 132.3, 134.7, 139.3, 164.3 (CO-amide), 169.3 (CO-amide), 192.5 (α-*CO*CON) ppm. Anal. Calcd for C_26_H_30_N_4_O_6_: C, 63.15; H, 6.11; N, 11.33. Found: C, 63.27; H, 5.98; N, 11.49.

### 1, 1'-(Piperazine-1,4-diyl)*bis*[2-(2-acetamidophenyl)ethane-1,2-dione] 7d (^1^H NMR and ^13^C NMR attached as a supporting information; Additional file 4)

White solid; mp 118-120°C (dec.); yield (69% method A); (93% method B); IR (KBr, cm^−1^): 3364 (NH), 1712 (α-CO), 1644 (CO-amide), 1580 (CO-amide). ^1^H-NMR (DMSO-d_6_): δ = 2.13 (s, 3H, COCH_3_), 2.18 (s, 3H, COCH_3_), 3.41 (m, 2H, CH_2_), 3.55 (m, 2H, CH_2_), 3.64 (m, 2H, CH_2_), 3.77 (m, 2H, CH_2_), 7.29 (m, 2H, ArH), 7.75 (m, 4H, ArH), 8.17 (d, *J =* 8.08 Hz, 1H, ArH), 8.31 (d, *J =* 8.08 Hz, 1H, ArH), 10.73 (s, 1H, NH), 10.83 (s, 1H, NH) ppm; ^13^C-NMR (DMSO-d_6_): δ = 25.1, 25.2, 41.1, 41.4, 45.4, 46.0, 121.8, 121.9, 122.1, 124.2, 124.2, 124.4, 133.4, 133.7, 136.5, 136.7, 140.6, 141.0, 164.6 (CO-amide), 164.7 (CO-amide), 169.9 (CO-amide), 193.4 (α-*CO*CON), 193.9 (C = O) ppm. Anal. Calcd for C_24_H_24_N_4_O_6_: C, 62.06; H, 5.21; N, 12.06. Found: C, 62.31; H, 5.11; N, 12.29.

### *N, N'*-(Cyclohexane-1,4-diyl)*bis(*2-(2-acetamidophenyl)-2-oxoacetamide] 7e (^1^H NMR and ^13^C NMR attached as a supporting information; Additional file 4)

Off White solid; mp 249-250°C; yield (68% method A); (89% method B); IR (KBr, cm^−1^): 3269 (NH), 1686 (α-CO), 1645 (CO-amide), 1588 (CO-amide). ^1^H-NMR (DMSO-d_6_): δ = 1.45 (m, 4H, CH_2_), 1.89 (m, 4H, CH_2_), 2.08 (s, 6H, 2 COCH_3_), 3.67 (m, 2H, CH), 7.26 (t, *J =* 7.36 Hz, 2H, 2ArH), 7.63 (m, 4H, 2NH, 2ArH), 7.97 (d, *J =* 8.08 Hz, 2H, ArH), 8.69 (d, *J =* 8.08 Hz, 2H, 2ArH), 10.64 (s, 2H, 2NH) ppm; ^13^C-NMR (DMSO-d_6_): δ = 24.7, 24.8, 31.1, 47.8, 121.8, 121.9, 123.7, 124.1, 132.3, 132.5, 134.7, 135.1, 139.5, 163.8 (CO-amide), 169.4 (CO-amide), 192.7 (α-*CO*CON) ppm. Anal. Calcd for C_26_H_28_N_4_O_6_: C, 63.40; H, 5.73; N, 11.38. Found: C, 63.28; H, 5.81; N, 11.49.

### *N, N'*-(Ethane-1,2-diyl)*bis*[2-(2-propionamidophenyl)-2-oxoacetamide] 8a (^1^H NMR and ^13^C NMR attached as a supporting information; Additional file 5)

White solid; mp 204-206°C (dec.); yield (70% method A); (91% method B); IR (KBr, cm^−1^): 3291 (NH), 1697 (α-CO), 1667 (CO-amide), 1607 (CO-amide). ^1^H-NMR (DMSO-d_6_): δ = 1.09 (t, *J =* 7.32 Hz, 6H, 2CH_2_CH_3_), 2.37 (q, *J =* 7.33 Hz, 4H, 2CH_2_CH_3_), 3.38 (m, 4H, 2CH_2_), 7.21 (t, *J =* 8.04 Hz, 2H, ArH), 7.62 (t, *J =* 7.32 Hz, 2H, 2NH), 7.69 (d, *J =* 7.32 Hz, 2H, ArH), 7.92 (d, *J =* 8.80 Hz, 2H, ArH), 8.88 (brs, 2H, ArH), 10.60 (s, 2H, 2NH) ppm; ^13^C-NMR (DMSO-d_6_): δ = 9.9, 30.4, 39.4, 121.8, 123.8, 124.0, 132.5, 134.7, 139.2, 164.3 (CO-amide), 172.9 (CO-amide), 191.9 (α-*CO*CON) ppm. Anal. Calcd for C_24_H_26_N_4_O_6_: C, 61.79; H, 5.62; N, 12.01; O, 20.58. Found: C, 62.00; H, 5.51; N, 12.39.

### 1, 1'-(Piperazine-1,4-diyl)bis[2-(2-propionamidophenyl)ethane-1,2-dione] 8d (^1^H NMR and ^13^C NMR attached as a supporting information; Additional file 5)

White solid; mp 221-223°C; yield (72% method A); (93% method B); IR (KBr, cm^−1^): 3318 (NH), 1701 (α-CO), 1644 (CO-amide), 1581 (CO-amide). ^1^H-NMR (DMSO-d_6_): δ = 1.13 (m, 4H, 2CH_2_CH_3_), 2.48 (m, 4H, 2CH_2_CH_3_), 3.40 (m, 2H, CH_2_), 3.55 (m, 2H, CH_2_), 3.64 (m, 2H, CH_2_), 3.78 (m, 2H, CH_2_), 7.29 (m, 2H, ArH), 7.75 (m, 4H, ArH), 8.30 (d, *J =* 8.08 Hz, 1H, ArH), 8.44 (d, *J =* 8.04 Hz, 1H, ArH), 10.80 (s, 1H, NH), 10.90 (s, 1H, NH) ppm; ^13^C-NMR (DMSO-d_6_): δ = 9.8, 9.8, 30.8, 30.9, 41.1, 41.4, 45.4, 46.0, 120.4, 121.5, 121.7, 124.0, 124.1, 133.7, 134.0, 136.7, 136.9, 141.1, 141.5, 164.7 (CO-amide), 164.7 (CO-amide), 173.3, 193.9 (α-*CO*CON), 194.3 (α-*CO*CON) ppm. Anal. Calcd for C_26_H_28_N_4_O_6_; C, 63.40: H, 5.73; N, 11.38. Found: C, 63.31; H, 5.81; N, 11.19.

### Synthesis of methyl 2-(2-acetamidophenyl)-2-oxoacetate (9a) and methyl 2-oxo-2-(2-propionamidophenyl)acetate (9b) using microwave irradiation

*N*-Acylisatin **1** or **2** (2 mmol) was dissolved in methanol (10 mL) and the reaction was microwave irradiated using a multimode reactor (Synthos 3000 Aton Paar, GmbH, 1400 W maximum magnetron) for 3 min at 80°C and hold at the same temperature for 2 min at 200 W. Cooling was accomplished by a fan (5 min) and the desired product was obtained after cooling with an excellent yield without further recrystallization.

### Methyl 2-(2-acetamidophenyl)-2-oxoacetate 9a (^1^H NMR and ^13^C NMR attached as a supporting information; Additional file 6)

The product was obtained as yellow needles from methanol; mp 104-105°C; yield 91%. IR (KBr, cm^−1^): 3220.99 (NH), 1746.58 (CO-ester), 1696.22 (α-CO), 1656.79 (CO-amide). ^1^H NMR (CDCl_3_): δ = 2.25 (s, 3H, COCH_3_), 3.98 (s, 3H, COOCH_3_), 7.13 (t, *J =* 7.36 Hz, 1H, Ar), 7.62-7.68 (m, 2H, Ar), 8.78(d, *J =* 8.08 Hz, 1H, Ar), 11.06 (s, 1H, NH) ppm. ^13^C NMR (CDCl_3_): δ = 25.6, 53.1, 117.1, 120.8, 122.6, 133.6, 137.3, 142.8, 142.8, 164.0 (CO-amide), 169.6 (CO-ester), 190.3 (α-*CO*CON) ppm. Anal. Calcd for C_11_H_11_NO_4_: C 59.73, H 5.01, N 6.33. Found C 60.00, H 5.18, N 6.44.

### Methyl 2-(2-propionamidophenyl)-2-oxo-acetate 9b (^1^H NMR and ^13^C NMR attached as a supporting information; Additional file 7)

The product was obtained as yellow needles from methanol; mp 68-70°C; yield 89%. IR (KBr, cm^−1^): 3220.99 (NH), 1746.58 (CO-ester), 1696.22 (α-CO), 1656.79 (CONH). ^1^H NMR (CDCl_3_): δ 1.28 (t, *J =* 7.32 Hz, 3H, CH_2_CH_3_), 2.48 (q, *J =* 7.32 Hz, 2H, COCH_2_CH_3_), 3.98 (s, 3H, COOCH_3_), 7.13 (t, *J =* 8.04 Hz, 1H, Ar), 7.63-7.68 (m, 2H, Ar), 8.82 (d, *J =* 8.08 Hz, 1H, Ar), 11.10 (s, 1H, NH) ppm; ^13^C NMR (CDCl_3_): δ = 9.5, 31.8, 53.1, 117.1, 120.8, 122.5, 133.7, 137.3, 142.9, 164.0 (CO-amide), 173.4 (CO-ester), 190.4 (α-*CO*CON) ppm. Anal. Calcd for C_12_H_13_NO_4_: C, 61.27; H, 5.57; N, 5.95. Found C 61.05, H 5.71, N 6.04.

## Conclusions

In conclusion, we have demonstrated that microwave irradiation could be employed efficiently for the synthesis of biologically important α-ketoamide and *bis*-(α-ketoamide) derivatives. The microwave irradiation showed more advantageous over the classical method with regard to reaction time, solvent quantity, and product yield in almost every case. Reaction of *N*-acylisatin with methanol under microwave irradiation afforded the α-phenylglyoxyl methyl ester derivatives with an excellent yield and purity. Aminolysis of phenylglyoxyl ester derivatives with piperidine and morpholine under microwave irradiation afford the same α-ketoamide derivatives as obtained from direct aminolysis of *N*-acylisatin with amine.

## Competing interests

The authors declare that they have no competing interests.

## Authors’ contributions

HAG and H-KF carried out X-ray method and the characterization; MRHS, SNK, and AEF carried out the synthesis and designed the proposed methods and analyzed the data statistically together. All authors read and approved the final manuscript.

## Supplementary Material

Additional file 1 ^**1**^**H NMR spectra of compound of compound 4a. **^13^C NMR spectra of compound of compound 4a. ^1^H NMR spectra of compound of compound 4b. ^13^C NMR spectra of compound of compound 4b. ^1^H NMR spectra of compound of compound 4c. ^13^C NMR spectra of compound of compound 4c. Click here for file

Additional file 2 ^**1**^**H NMR spectra of compound of compound 5a. **^13^C NMR spectra of compound of compound 5a. ^1^H NMR spectra of compound of compound 5b. ^13^C NMR spectra of compound of compound 5b. ^1^H NMR spectra of compound of compound 5c. ^13^C NMR spectra of compound of compound 5c. Click here for file

Additional file 3 ^**1**^**H NMR spectra of compound of compound 7a. **^13^C NMR spectra of compound of compound 7a. ^1^H NMR spectra of compound of compound 7b. ^13^C NMR spectra of compound of compound 7b. Click here for file

Additional file 4 ^**1**^**H NMR spectra of compound of compound 7d. **^13^C NMR spectra of compound of compound 7d. ^1^H NMR spectra of compound of compound 7e. ^13^C NMR spectra of compound of compound 7e. ^1^H NMR spectra of compound of compound 7c. ^13^C NMR spectra of compound of compound 7c. Click here for file

Additional file 5 ^**1**^**H NMR spectra of compound of compound 8a. **^13^C NMR spectra of compound of compound 8a. ^1^H NMR spectra of compound of compound 8d. ^13^C NMR spectra of compound of compound 8d. Click here for file

Additional file 6 ^**1**^**H NMR spectra of compound of compound 9a. **^13^C NMR spectra of compound of compound 9a. Click here for file

Additional file 7 ^**1**^**H NMR spectra of compound of compound 9b. **^13^C NMR spectra of compound of compound 9b. Click here for file
