# Supplementary material for: Microwave irradiation: synthesis and characterization of α-ketoamide and bis (α-ketoamide) derivatives via the ring opening of N-acetylisatin
Source: Chem Cent J. 2014 Apr 28;8:27. doi: 10.1186/1752-153X-8-27 (PMC4021159; doi:10.1186/1752-153X-8-27)
Supplement: Additional file 1 — 1H NMR spectra of compound of compound 4a. 13C NMR spectra of compound of compound 4a. 1H NMR spectra of compound of compound 4b. 13C NMR spectra of compound of compound 4b. 1H NMR spectra of compound of compound 4c. 13C NMR spectra of compound of compound 4c. [file 1752-153X-8-27-S1.pdf]

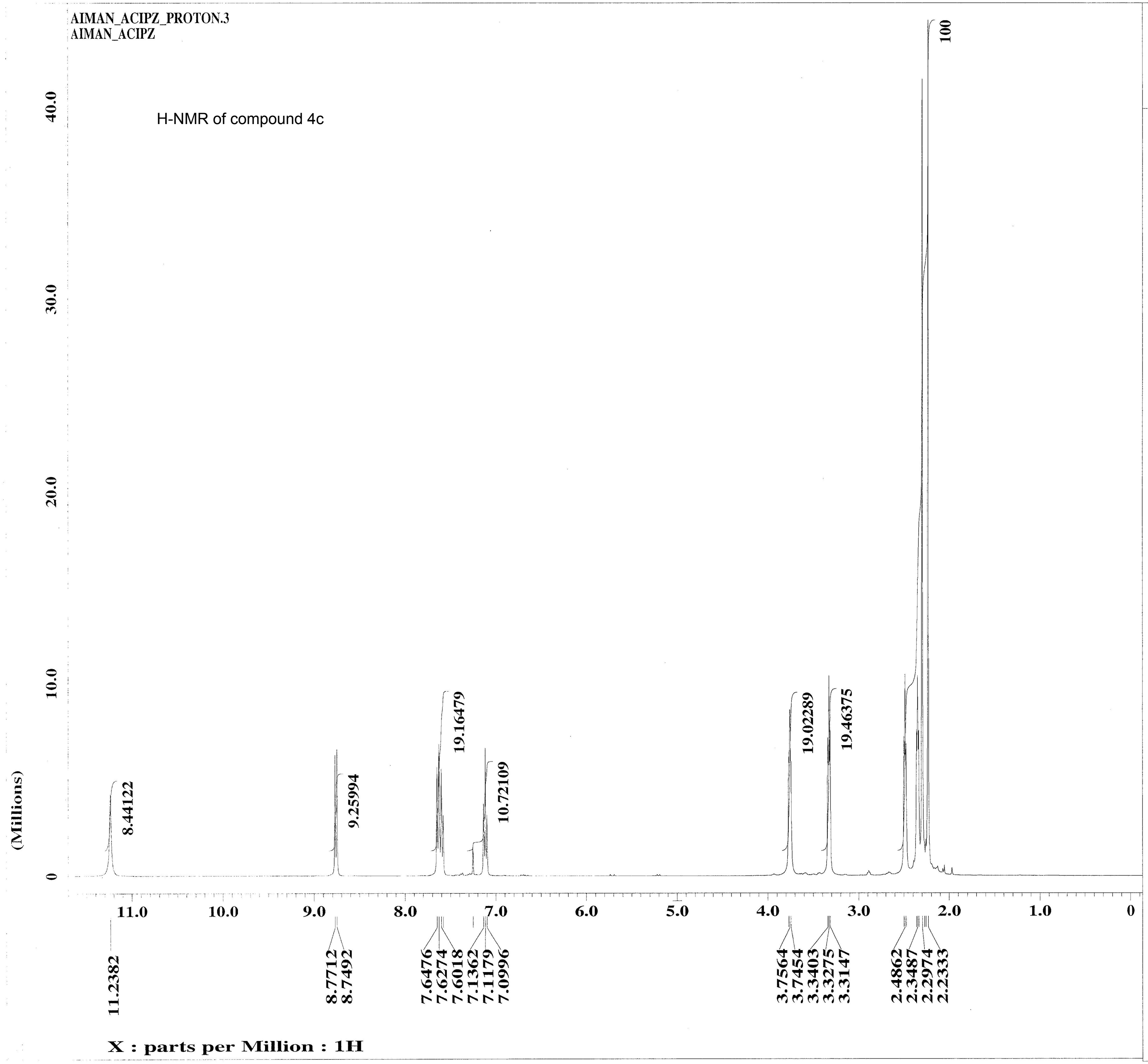


```
---- ACQUISITION PARAMETERS ----
File Name          = AIMAN_ACIPZ_PROTON.3
Author             = DR. M. MARASHDAH
Sample ID          = AIMAN_ACIPZ
Content            = AIMAN_ACIPZ
Creation Date      =  3-APR-2013 09:39:06

Revision Date      = 13-APR-2013 10:43:01
Spec Site          = ECP400

Spec Type          = DELTA_NMR
Data Format        = 1D COMPLEX
Dimensions         = X
Dim Title          = 1H
Dim Size           = 16384
Dim Units          = [ppm]
Experiment         = single_pulse.exp
Field_strength     = 9.389766[T]
X_domain           = 1H
X_freq             = 399.7841973[MHz]
X_offset           = 5[ppm]
X_sweep            = 12.00480192[kHz]
X_points           = 16384
X_resolution       = 0.73275969[Hz]
Recvr_gain         = 13
Filter_mode        = BUTTERWORTH
X_prescans         = 0
Scans              = 8
Irr_noise          = WALTZ
Irr_pwidth         = 50[us]
Relaxation_delay= 4[s]
Solvent            = CHLOROFORM-D
Temp_get           = 22.4[dC]
Spin_get           = 12[Hz]
Probe_id           = 2564
```

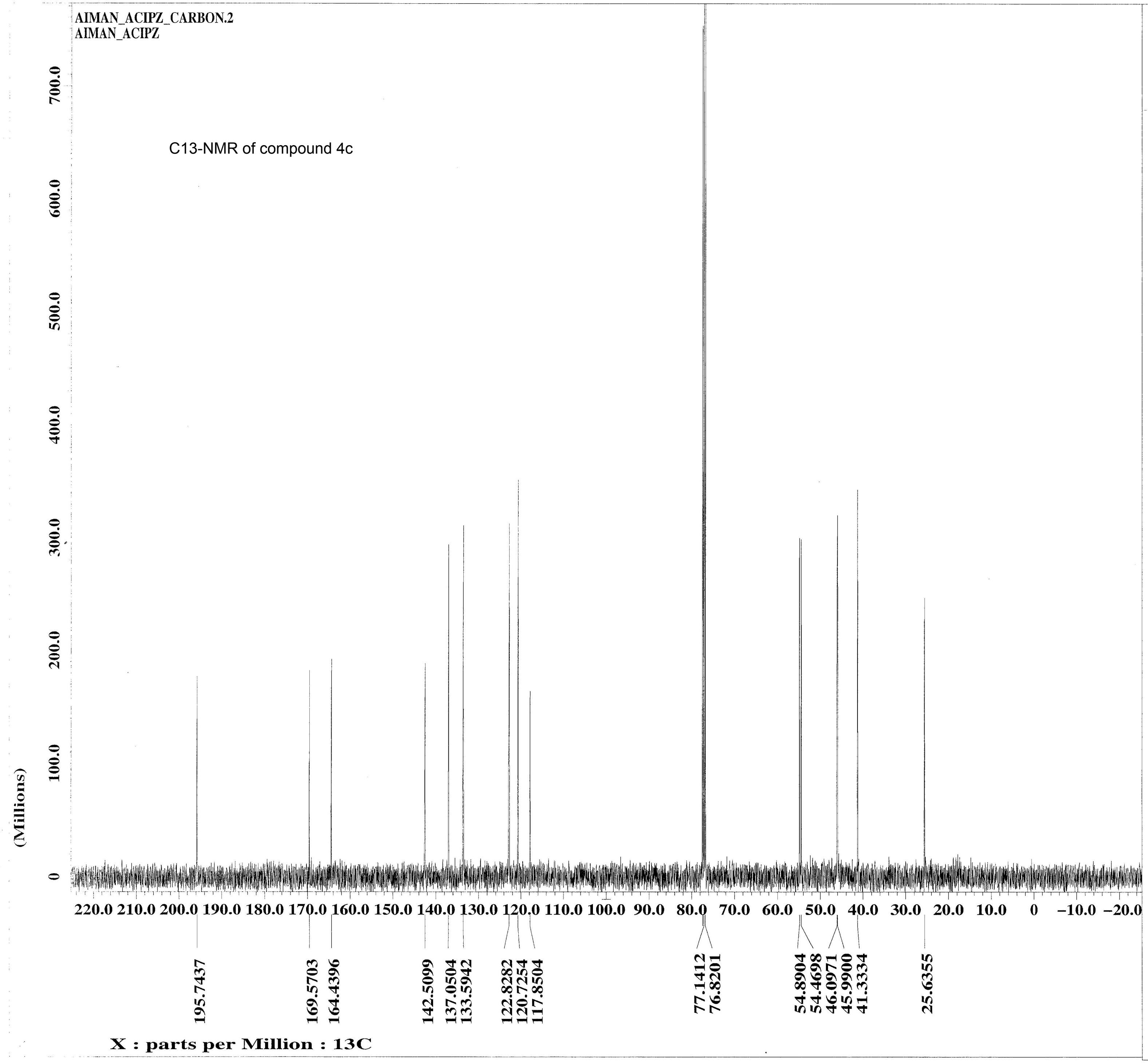

---- ACQUISITION PARAMETERS ----

File Name	= AIMAN_ACIPZ_CARBON.2
Author	= DR. M. MARASHDAH
Sample ID	= AIMAN_ACIPZ
Content	= AIMAN_ACIPZ
Creation Date	= 3-APR-2013 09:48:04
Revision Date	= 3-APR-2013 10:48:27
Spec Site	= ECP400
Spec Type	= DELTA_NMR
Data Format	= 1D COMPLEX
Dimensions	= X
Dim Title	= 13C
Dim Size	= 32768
Dim Units	= [ppm]
Experiment	= single_pulse_dec
Field_strength	= 9.389766[T]
X_domain	= 13C
X_freq	= 100.53535686[MHz]
X_offset	= 100[ppm]
X_sweep	= 25.18891688[kHz]
X_points	= 32768
X_resolution	= 0.7687282[Hz]
Recvr_gain	= 29
Filter_mode	= BUTTERWORTH
X_prescans	= 4
Scans	= 204
Irr_domain	= 1H
Irr_offset	= 5.0[ppm]
Irr_noise	= WALTZ
Irr_pwidth	= 50[us]
Relaxation_delay	= 1[s]
Solvent	= CHLOROFORM-D
Temp_get	= 23.4[dC]
Spin_get	= 18[Hz]
Probe_id	= 2564

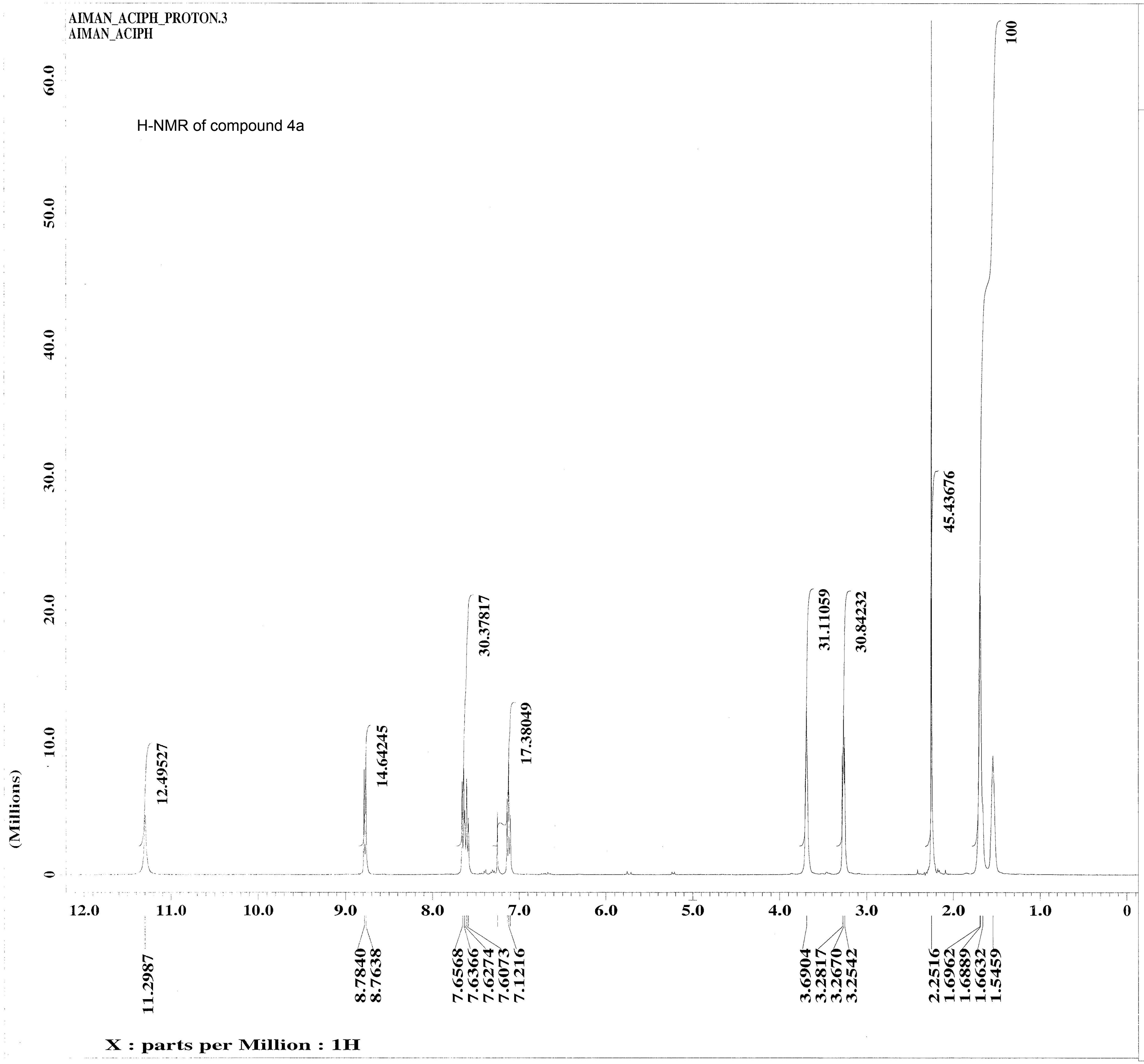

---- ACQUISITION PARAMETERS ----

File Name = AIMAN_ACIPH_PROTON.3
Author = DR. M. MARASHDAH
Sample ID = AIMAN_ACIPH
Content = AIMAN_ACIPH
Creation Date = 1-APR-2013 10:48:11

Revision Date = 1-APR-2013 11:39:46
Spec Site = ECP400

Spec Type = DELTA_NMR
Data Format = 1D COMPLEX
Dimensions = X
Dim Title = 1H
Dim Size = 16384
Dim Units = [ppm]
Experiment = single_pulse.exp
Field_strength = 9.389766[T]
X_domain = 1H
X_freq = 399.7841973[MHz]
X_offset = 5[ppm]
X_sweep = 12.00480192[kHz]
X_points = 16384
X_resolution = 0.73275969[Hz]
Recvr_gain = 17
Filter_mode = BUTTERWORTH
X_prescans = 0
Scans = 8
Irr_noise = WALTZ
Irr_pwidth = 50[us]
Relaxation_delay= 4[s]
Solvent = CHLOROFORM-D
Temp_get = 21.7[dC]
Spin_get = 16[Hz]
Probe_id = 2564

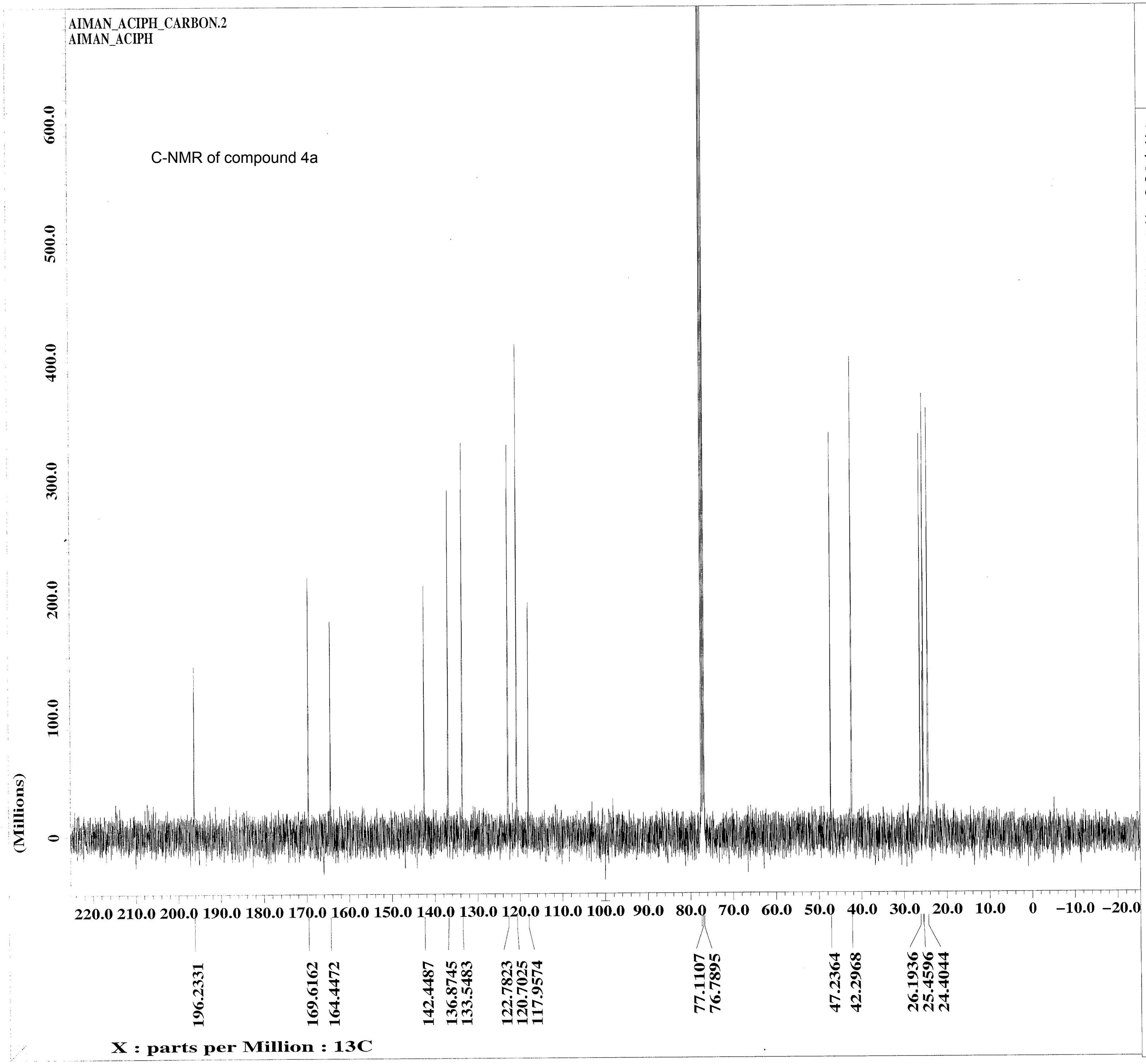

JEOL

---- ACQUISITION PARAMETERS ----

File Name	= AIMAN_ACIPH_CARBON.2
Author	= DR. M. MARASHDAH
Sample ID	= AIMAN_ACIPH
Content	= AIMAN_ACIPH
Creation Date	= 1-APR-2013 11:07:35
Revision Date	= 1-APR-2013 11:42:21
Spec Site	= ECP400
Spec Type	= DELTA_NMR
Data Format	= 1D COMPLEX
Dimensions	= X
Dim Title	= 13C
Dim Size	= 32768
Dim Units	= [ppm]
Experiment	= single_pulse_dec
Field_strength	= 9.389766[T]
X_domain	= 13C
X_freq	= 100.53535686[MHz]
X_offset	= 100[ppm]
X_sweep	= 25.18891688[kHz]
X_points	= 32768
X_resolution	= 0.7687282[Hz]
Recvr_gain	= 29
Filter_mode	= BUTTERWORTH
X_prescans	= 4
Scans	= 475
Irr_domain	= 1H
Irr_offset	= 5.0[ppm]
Irr_noise	= WALTZ
Irr_pwidth	= 50[us]
Relaxation_delay	= 1[s]
Solvent	= CHLOROFORM-D
Temp_get	= 22.7[dC]
Spin_get	= 16[Hz]
Probe_id	= 2564

H-NMR of compound 4b

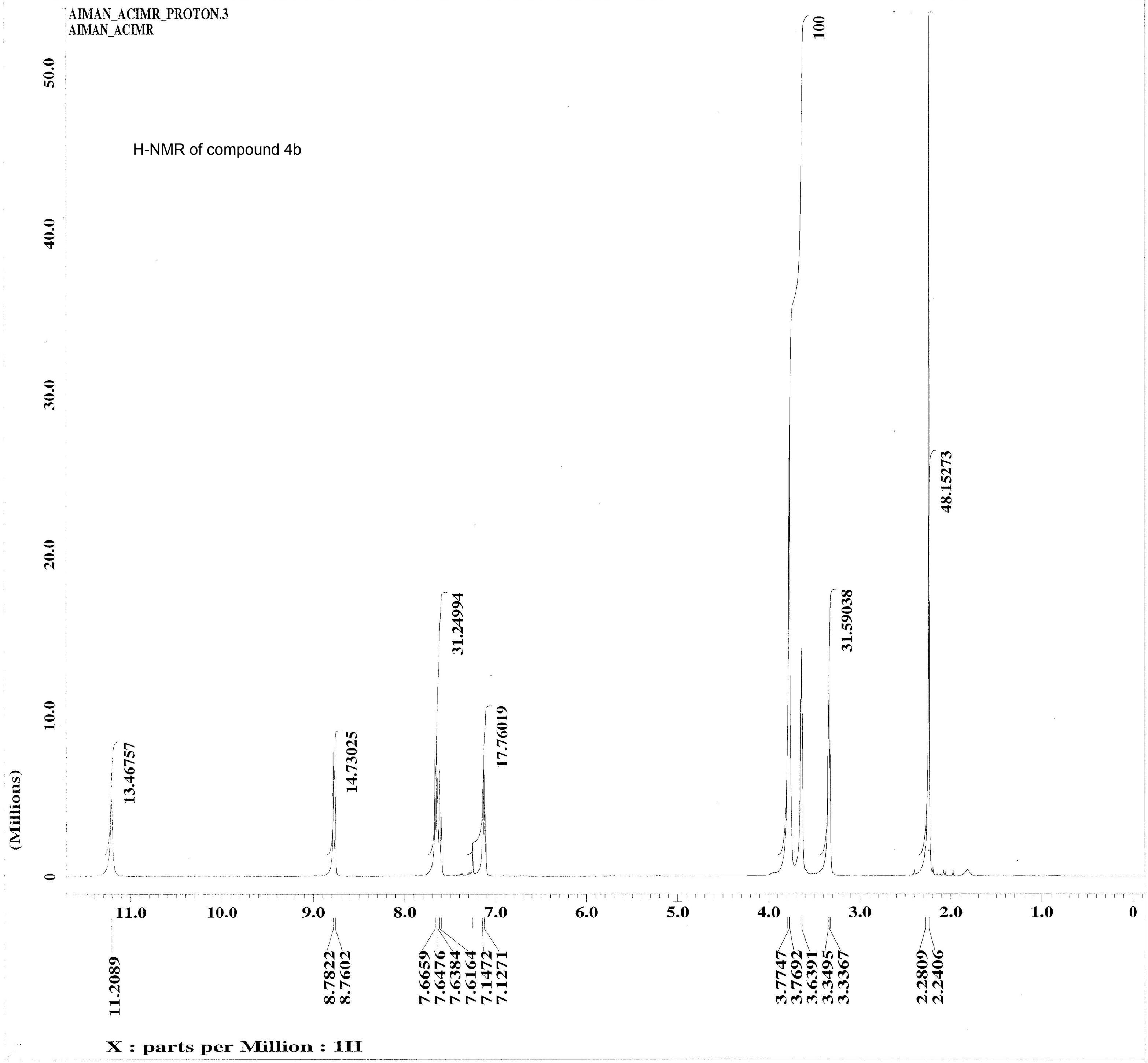


```
---- ACQUISITION PARAMETERS ----
File Name          = AIMAN_ACIMR_PROTON.3
Author             = DR. M. MARASHDAH
Sample ID          = AIMAN_ACIMR
Content            = AIMAN_ACIMR
Creation Date      =  3-APR-2013 09:03:51

Revision Date      = 13-APR-2013 10:44:24
Spec Site          = ECP400

Spec Type          = DELTA_NMR
Data Format        = 1D COMPLEX
Dimensions         = X
Dim Title          = 1H
Dim Size           = 16384
Dim Units          = [ppm]
Experiment         = single_pulse.exp
Field_strength     = 9.389766[T]
X_domain           = 1H
X_freq             = 399.7841973[MHz]
X_offset           = 5[ppm]
X_sweep            = 12.00480192[kHz]
X_points           = 16384
X_resolution       = 0.73275969[Hz]
Recvr_gain         = 14
Filter_mode        = BUTTERWORTH
X_prescans         = 0
Scans              = 8
Irr_noise          = WALTZ
Irr_pwidth         = 50[us]
Relaxation_delay= 4[s]
Solvent            = CHLOROFORM-D
Temp_get           = 21.9[dC]
Spin_get           = 16[Hz]
Probe_id           = 2564
```

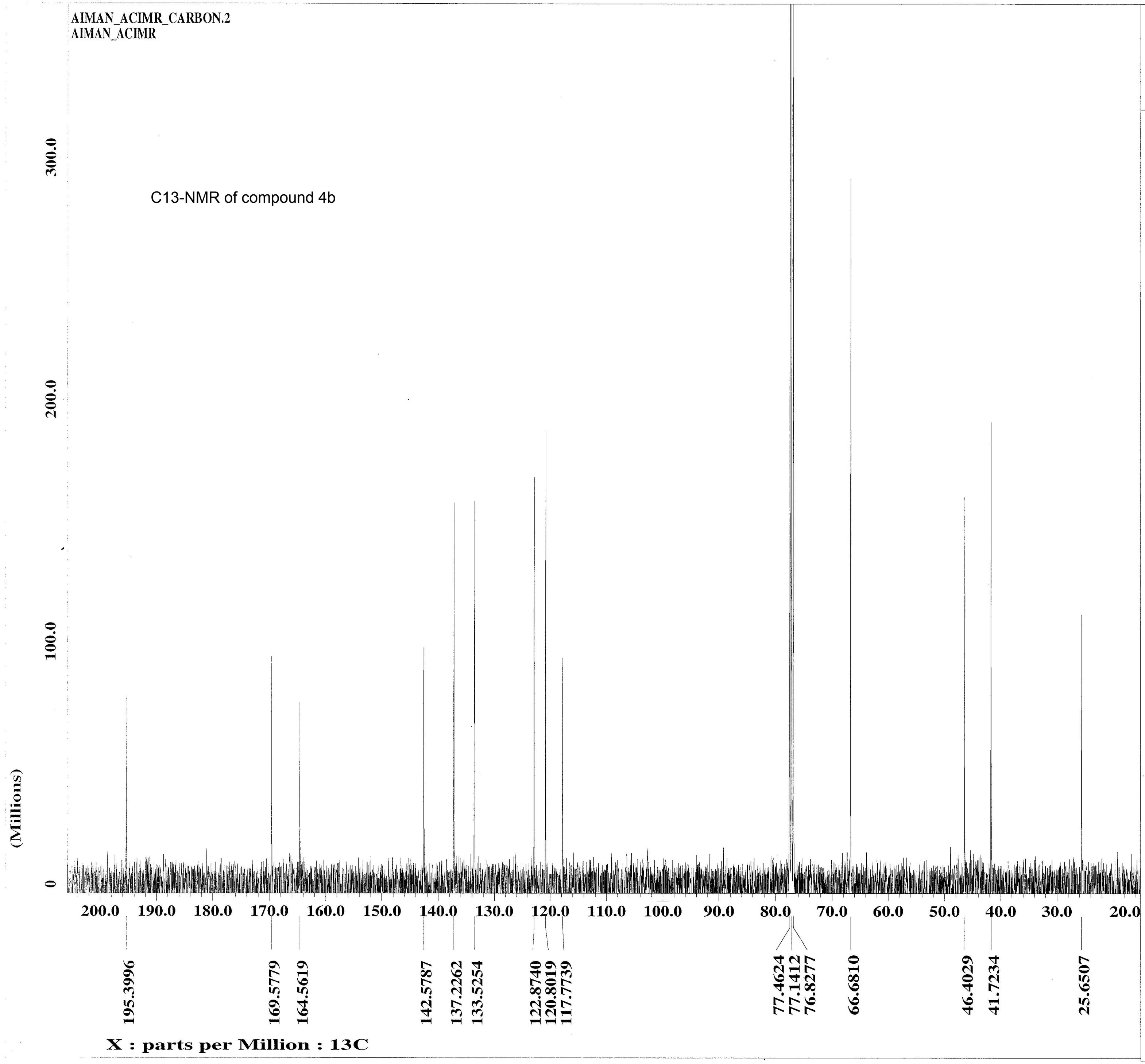

JEOL

---- ACQUISITION PARAMETERS ----

File Name	= AIMAN_ACIMR_CARBON.2
Author	= DR. M. MARASHDAH
Sample ID	= AIMAN_ACIMR
Content	= AIMAN_ACIMR
Creation Date	= 3-APR-2013 09:09:27
Revision Date	= 3-APR-2013 09:50:04
Spec Site	= ECP400
Spec Type	= DELTA_NMR
Data Format	= 1D COMPLEX
Dimensions	= X
Dim Title	= 13C
Dim Size	= 32768
Dim Units	= [ppm]
Experiment	= single_pulse_dec
Field_strength	= 9.389766[T]
X_domain	= 13C
X_freq	= 100.53535686[MHz]
X_offset	= 100[ppm]
X_sweep	= 25.18891688[kHz]
X_points	= 32768
X_resolution	= 0.7687282[Hz]
Recvr_gain	= 29
Filter_mode	= BUTTERWORTH
X_prescans	= 4
Scans	= 116.0
Irr_domain	= 1H
Irr_offset	= 5.0[ppm]
Irr_noise	= WALTZ
Irr_pwidth	= 50[us]
Relaxation_delay	= 1[s]
Solvent	= CHLOROFORM-D
Temp_get	= 22.8[dC]
Spin_get	= 16[Hz]
Probe_id	= 2564
