# Supplementary material for: Microwave irradiation: synthesis and characterization of α-ketoamide and bis (α-ketoamide) derivatives via the ring opening of N-acetylisatin
Source: Chem Cent J. 2014 Apr 28;8:27. doi: 10.1186/1752-153X-8-27 (PMC4021159; doi:10.1186/1752-153X-8-27)
Supplement: Additional file 2 — 1H NMR spectra of compound of compound 5a. 13C NMR spectra of compound of compound 5a. 1H NMR spectra of compound of compound 5b. 13C NMR spectra of compound of compound 5b. 1H NMR spectra of compound of compound 5c. 13C NMR spectra of compound of compound 5c. [file 1752-153X-8-27-S2.pdf]

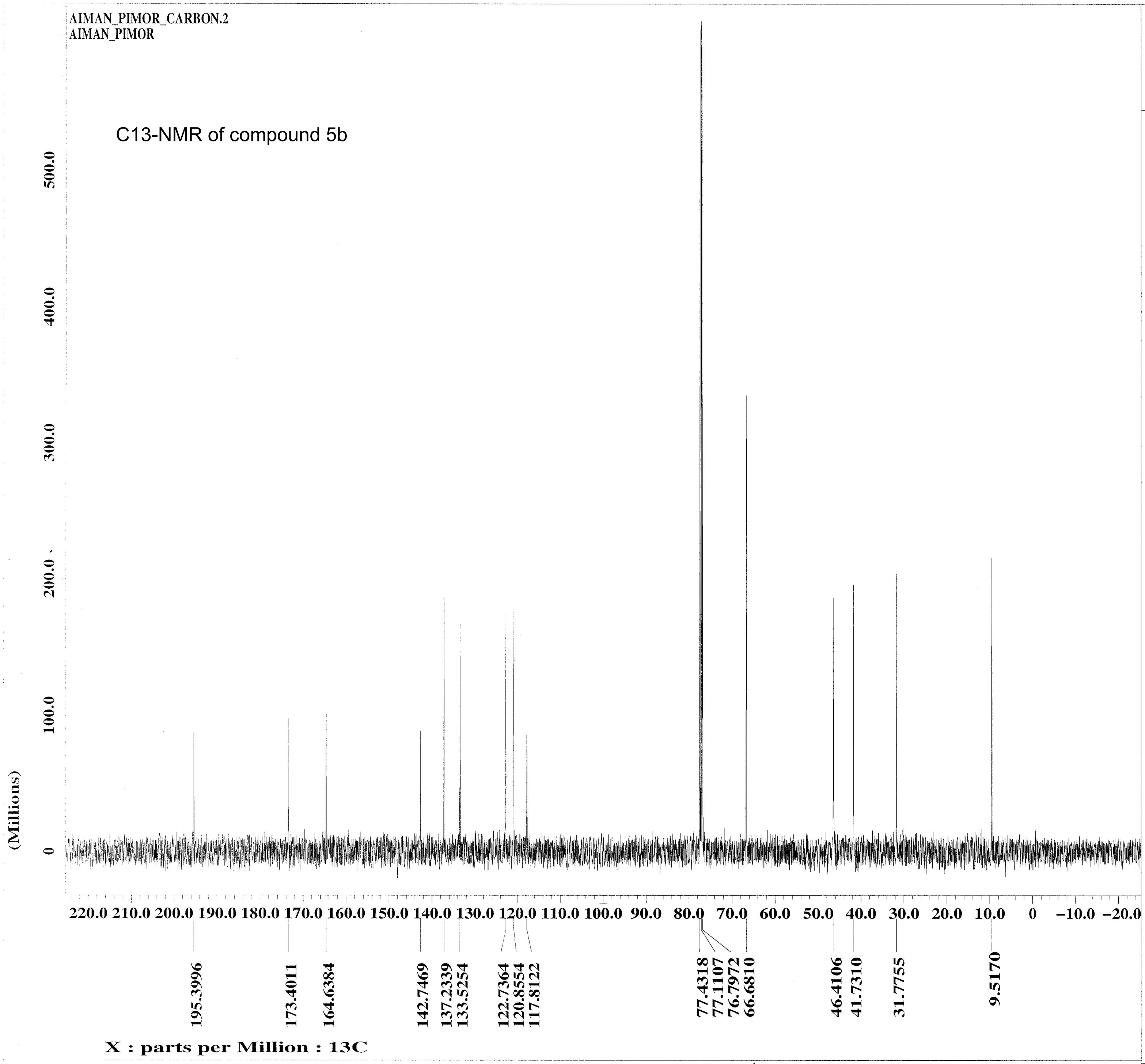

JEOL

```
---- ACQUISITION PARAMETERS ----
File Name          = AIMAN_PIMOR_CARBON.2
Author             = DR. M. MARASHDAH
Sample ID          = AIMAN_PIMOR
Content            = AIMAN_PIMOR
Creation Date      = 27-MAY-2013 12:52:38

Revision Date      = 27-MAY-2013 14:32:28
Spec Site          = ECP400

Spec Type          = DELTA_NMR
Data Format        = 1D COMPLEX
Dimensions         = X
Dim Title          = 13C
Dim Size           = 32768
Dim Units          = [ppm]
Experiment         = single_pulse_dec
Field_strength     = 9.389766[T]
X_domain           = 13C
X_freq             = 100.53535686[MHz]
X_offset           = 100[ppm]
X_sweep            = 25.18891688[kHz]
X_points           = 32768
X_resolution       = 0.7687282[Hz]
Recvr_gain         = 29
Filter_mode        = BUTTERWORTH
X_prescans         = 4
Scans              = 198
Irr_domain         = 1H
Irr_offset         = 5.0[ppm]
Irr_noise          = WALTZ
Irr_pwidth         = 50[us]
Relaxation_delay= 1[s]
Solvent            = CHLOROFORM-D
Temp_get           = 24[dC]
Spin_get           = 15[Hz]
Probe_id           = 2564
```

AIMAN_PRIMPZ_PROTON.3
AIMAN_PRIMPZ

H-NMR of compound 5c

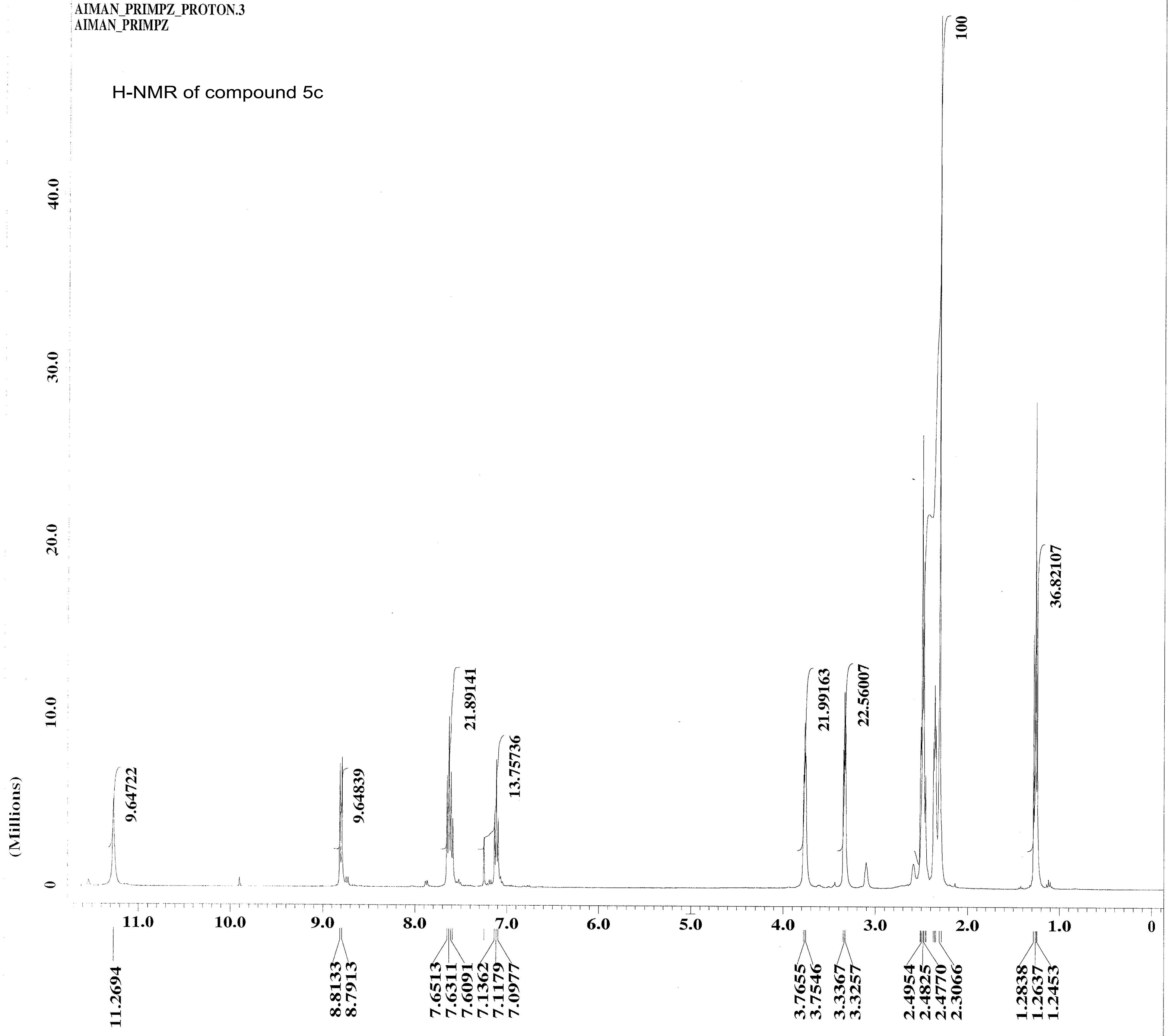

X : parts per Million : 1H

JEOL

---- ACQUISITION PARAMETERS ----

File Name	= AIMAN_PRIMPZ_PROTON.3
Author	= DR. M. MARASHDAH
Sample ID	= AIMAN_PRIMPZ
Content	= AIMAN_PRIMPZ
Creation Date	= 28-MAY-2013 12:58:55
Revision Date	= 29-MAY-2013 10:44:16
Spec Site	= ECP400
Spec Type	= DELTA_NMR
Data Format	= 1D COMPLEX
Dimensions	= X
Dim Title	= 1H
Dim Size	= 16384
Dim Units	= [ppm]
Experiment	= single_pulse.exp
Field_strength	= 9.389766[T]
X_domain	= 1H
X_freq	= 399.7841973[MHz]
X_offset	= 5[ppm]
X_sweep	= 12.00480192[kHz]
X_points	= 16384
X_resolution	= 0.73275969[Hz]
Recvr_gain	= 14
Filter_mode	= BUTTERWORTH
X_prescans	= 0
Scans	= 8
Irr_noise	= WALTZ
Irr_pwidth	= 50[us]
Relaxation_delay	= 4[s]
Solvent	= CHLOROFORM-D
Temp_get	= 22.7[dC]
Spin_get	= 15[Hz]
Probe_id	= 2564

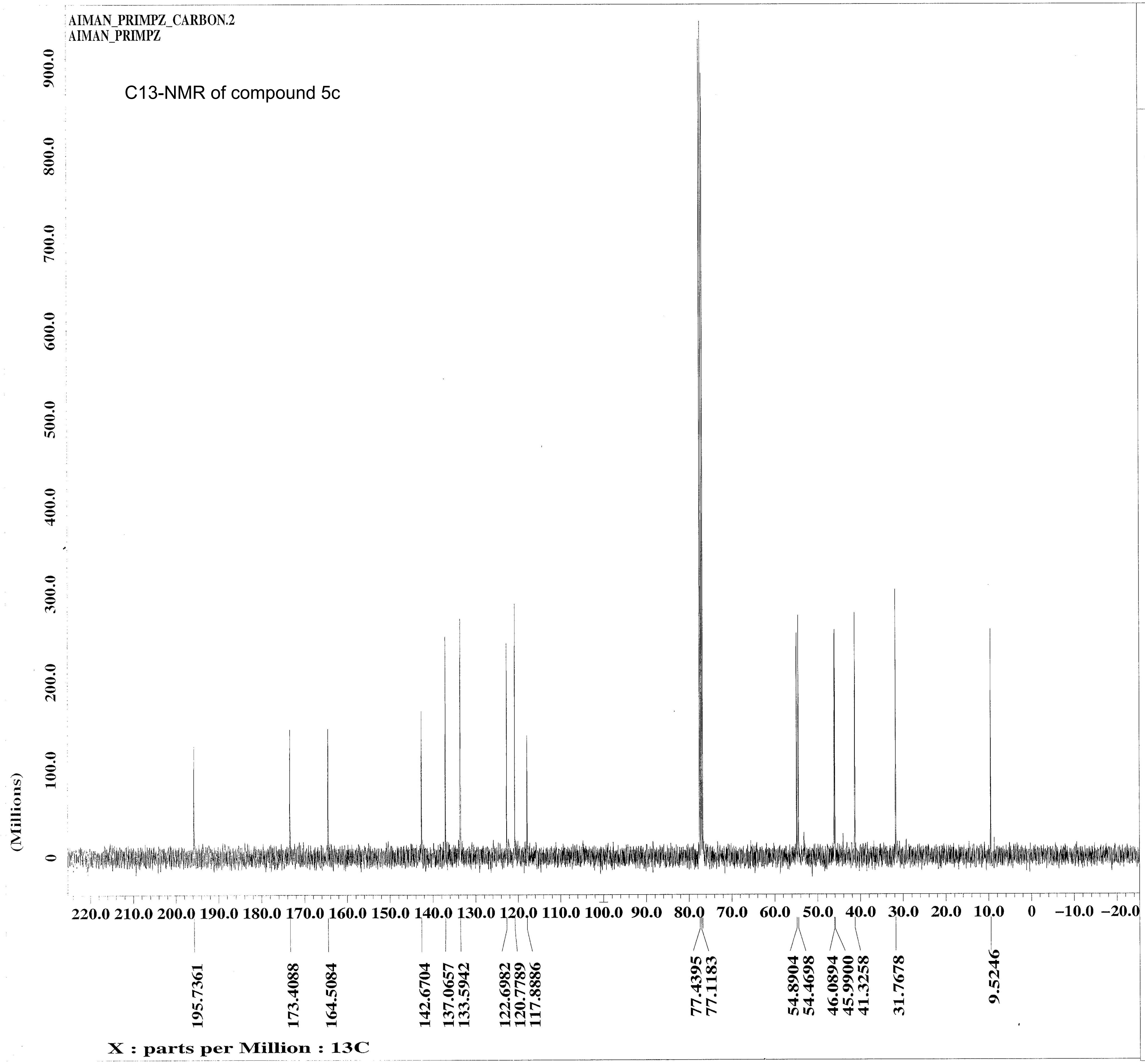

JEOL

---- ACQUISITION PARAMETERS ----

File Name	= AIMAN_PRIMPZ_CARBON.2
Author	= DR. M. MARASHDAH
Sample ID	= AIMAN_PRIMPZ
Content	= AIMAN_PRIMPZ
Creation Date	= 28-MAY-2013 13:10:51
Revision Date	= 29-MAY-2013 10:45:23
Spec Site	= ECP400
Spec Type	= DELTA_NMR
Data Format	= 1D COMPLEX
Dimensions	= X
Dim Title	= 13C
Dim Size	= 32768
Dim Units	= [ppm]
Experiment	= single_pulse_dec
Field_strength	= 9.389766[T]
X_domain	= 13C
X_freq	= 100.53535686[MHz]
X_offset	= 100[ppm]
X_sweep	= 25.18891688[kHz]
X_points	= 32768
X_resolution	= 0.7687282[Hz]
Recvr_gain	= 29
Filter_mode	= BUTTERWORTH
X_prescans	= 4
Scans	= 281
Irr_domain	= 1H
Irr_offset	= 5.0[ppm]
Irr_noise	= WALTZ
Irr_pwidth	= 50[us]
Relaxation_delay	= 1[s]
Solvent	= CHLOROFORM-D
Temp_get	= 23.8[dC]
Spin_get	= 14[Hz]
Probe_id	= 2564

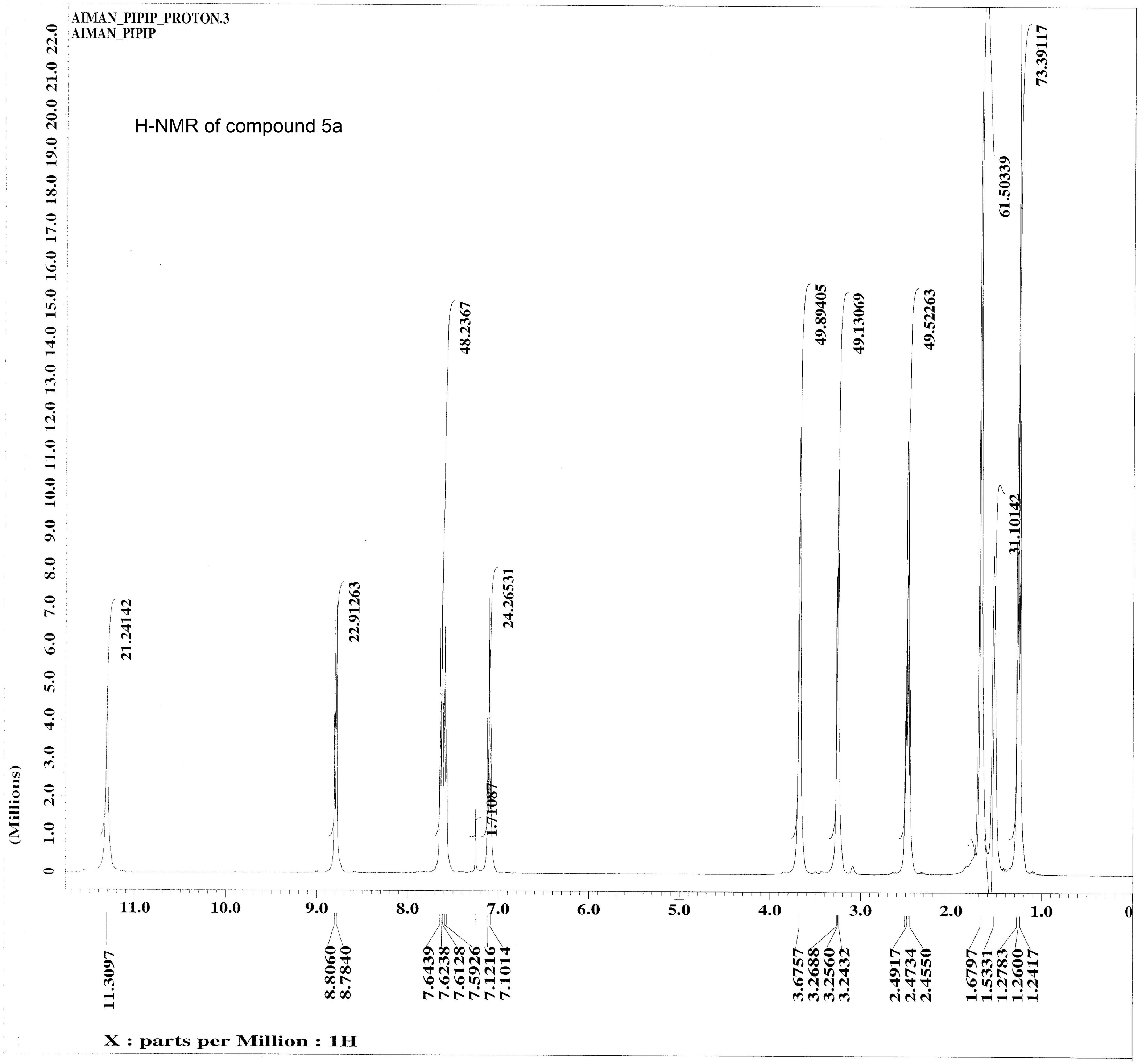

JEOL

---- ACQUISITION PARAMETERS ----

File Name	= AIMAN_PIPIP_PROTON.3
Author	= DR. M. MARASHDAH
Sample ID	= AIMAN_PIPIP
Content	= AIMAN_PIPIP
Creation Date	= 27-MAY-2013 12:58:57
Revision Date	= 27-MAY-2013 14:34:16
Spec Site	= ECP400
Spec Type	= DELTA_NMR
Data Format	= 1D COMPLEX
Dimensions	= X
Dim Title	= 1H
Dim Size	= 16384
Dim Units	= [ppm]
Experiment	= single_pulse.exp
Field_strength	= 9.389766[T]
X_domain	= 1H
X_freq	= 399.7841973[MHz]
X_offset	= 5[ppm]
X_sweep	= 12.00480192[kHz]
X_points	= 16384
X_resolution	= 0.73275969[Hz]
Recvr_gain	= 13
Filter_mode	= BUTTERWORTH
X_prescans	= 0
Scans	= 8
Irr_noise	= WALTZ
Irr_pwidth	= 50[us]
Relaxation_delay	= 4[s]
Solvent	= CHLOROFORM-D
Temp_get	= 22.9[dC]
Spin_get	= 15[Hz]
Probe_id	= 2564

AIMAN_PIPIP_CARBON.2
AIMAN_PIPIP

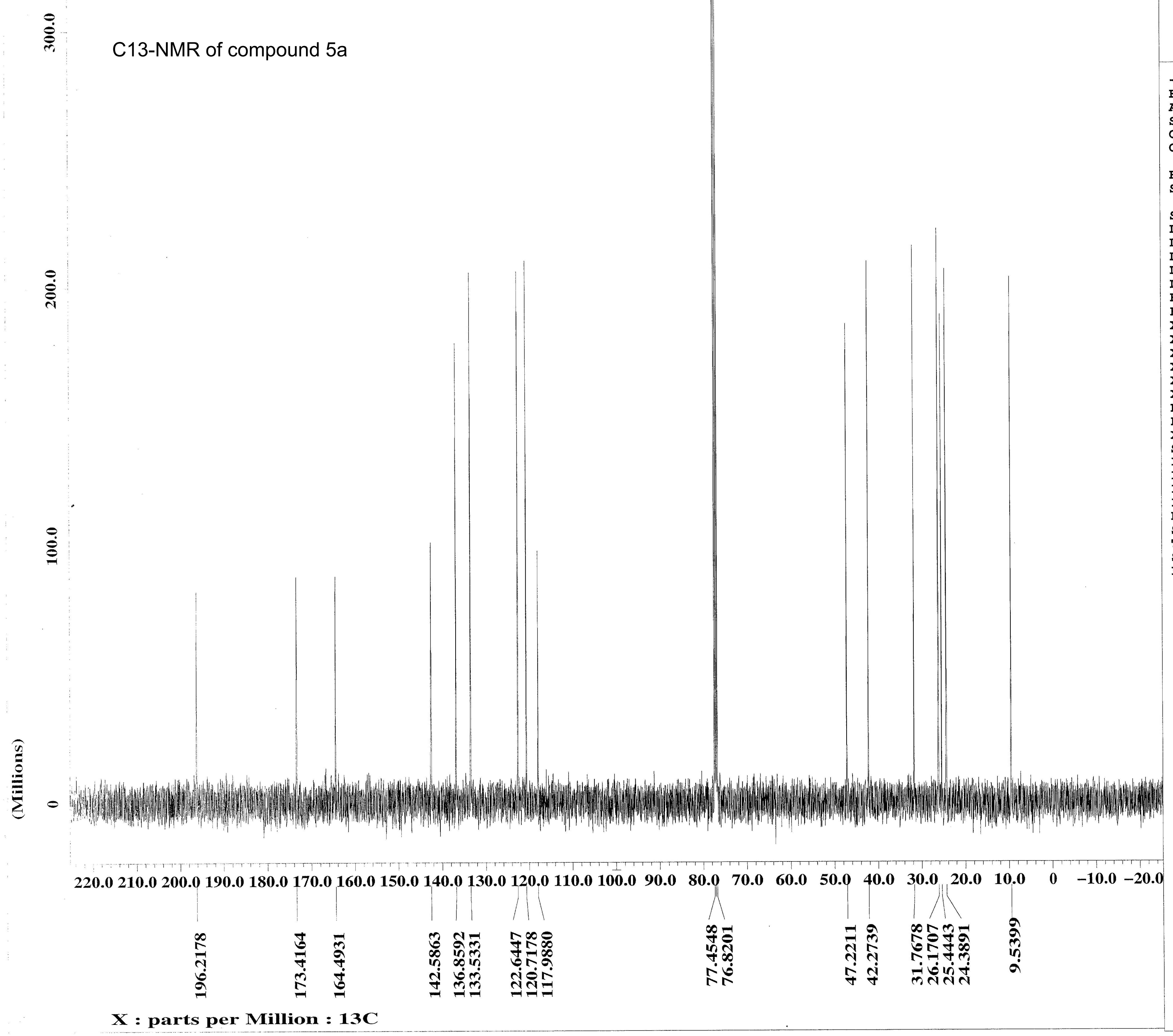

JEOL

---- ACQUISITION PARAMETERS ----

File Name	= AIMAN_PIPIP_CARBON.2
Author	= DR. M. MARASHDAH
Sample ID	= AIMAN_PIPIP
Content	= AIMAN_PIPIP
Creation Date	= 27-MAY-2013 13:04:35
Revision Date	= 27-MAY-2013 14:35:55
Spec Site	= ECP400
Spec Type	= DELTA_NMR
Data Format	= 1D COMPLEX
Dimensions	= X
Dim Title	= 13C
Dim Size	= 32768
Dim Units	= [ppm]
Experiment	= single_pulse_dec
Field_strength	= 9.389766[T]
X_domain	= 13C
X_freq	= 100.53535686[MHz]
X_offset	= 100[ppm]
X_sweep	= 25.18891688[kHz]
X_points	= 32768
X_resolution	= 0.7687282[Hz]
Recvr_gain	= 29
Filter_mode	= BUTTERWORTH
X_prescans	= 4
Scans	= 117
Irr_domain	= 1H
Irr_offset	= 5.0[ppm]
Irr_noise	= WALTZ
Irr_pwidth	= 50[us]
Relaxation_delay	= 1[s]
Solvent	= CHLOROFORM-D
Temp_get	= 23.9[dC]
Spin_get	= 15[Hz]
Probe_id	= 2564

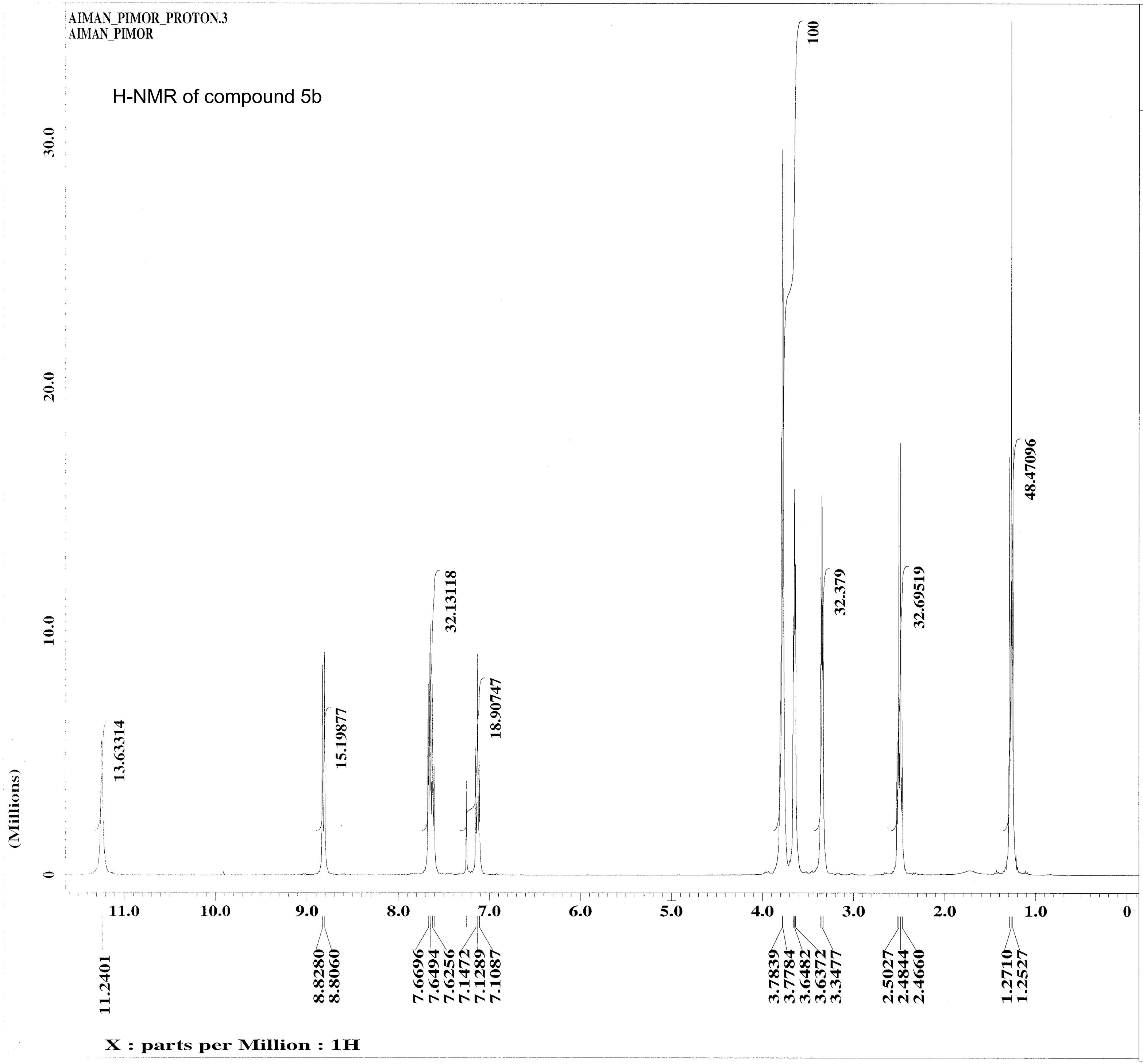

---- ACQUISITION PARAMETERS ----

File Name	= AIMAN_PIMOR_PROTON.3
Author	= DR. M. MARASHDAH
Sample ID	= AIMAN_PIMOR
Content	= AIMAN_PIMOR
Creation Date	= 27-MAY-2013 12:43:55
Revision Date	= 27-MAY-2013 14:31:14
Spec Site	= ECP400
Spec Type	= DELTA_NMR
Data Format	= 1D COMPLEX
Dimensions	= X
Dim Title	= 1H
Dim Size	= 16384
Dim Units	= [ppm]
Experiment	= single_pulse.exp
Field_strength	= 9.389766[T]
X_domain	= 1H
X_freq	= 399.7841973[MHz]
X_offset	= 5[ppm]
X_sweep	= 12.00480192[kHz]
X_points	= 16384
X_resolution	= 0.73275969[Hz]
Recvr_gain	= 15
Filter_mode	= BUTTERWORTH
X_prescans	= 0
Scans	= 8
Irr_noise	= WALTZ
Irr_pwidth	= 50[us]
Relaxation_delay	= 4[s]
Solvent	= CHLOROFORM-D
Temp_get	= 22.9[dC]
Spin_get	= 15[Hz]
Probe_id	= 2564
