# Supplementary material for: Microwave irradiation: synthesis and characterization of α-ketoamide and bis (α-ketoamide) derivatives via the ring opening of N-acetylisatin
Source: Chem Cent J. 2014 Apr 28;8:27. doi: 10.1186/1752-153X-8-27 (PMC4021159; doi:10.1186/1752-153X-8-27)
Supplement: Additional file 4 — 1H NMR spectra of compound of compound 7d. 13C NMR spectra of compound of compound 7d. 1H NMR spectra of compound of compound 7e. 13C NMR spectra of compound of compound 7e. 1H NMR spectra of compound of compound 7c. 13C NMR spectra of compound of compound 7c. [file 1752-153X-8-27-S4.pdf]

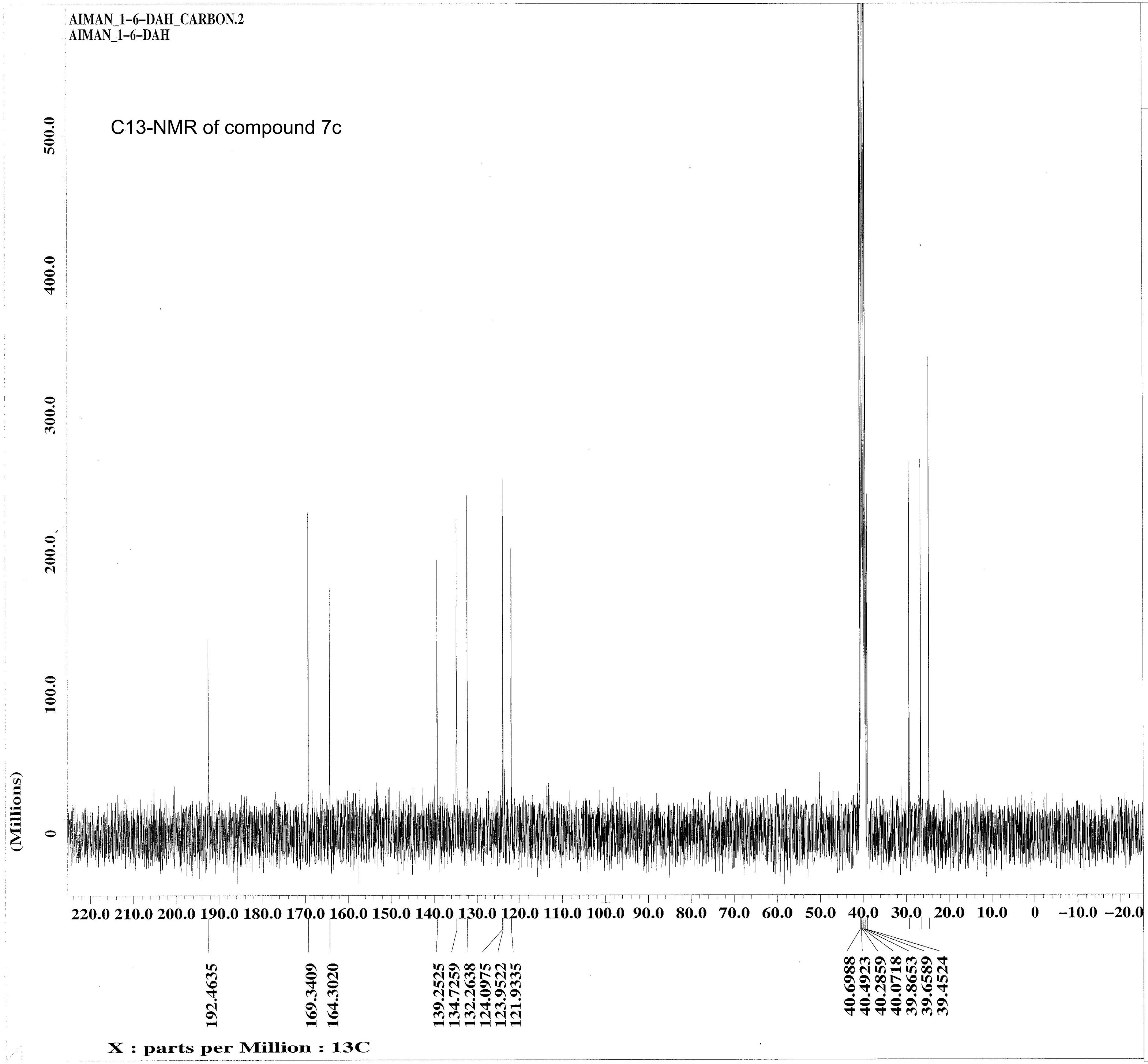

JEOL

```
---- ACQUISITION PARAMETERS ----
File Name          = AIMAN_1-6-DAH_CARBON.2
Author             = DR. M. MARASHDAH
Sample ID          = AIMAN_1-6-DAH
Content            = AIMAN_1-6-DAH
Creation Date      = 28-APR-2013 13:27:20

Revision Date      = 29-APR-2013 11:46:38
Spec Site          = ECP400

Spec Type          = DELTA_NMR
Data Format        = 1D COMPLEX
Dimensions         = X
Dim Title          = 13C
Dim Size           = 32768
Dim Units          = [ppm]
Experiment         = single_pulse_dec
Field_strength     = 9.389766[T]
X_domain           = 13C
X_freq             = 100.53535686[MHz]
X_offset           = 100[ppm]
X_sweep            = 25.18891688[kHz]
X_points           = 32768
X_resolution       = 0.7687282[Hz]
Recvr_gain         = 29
Filter_mode        = BUTTERWORTH
X_prescans         = 4
Scans              = 800
Irr_domain         = 1H
Irr_offset         = 5.0[ppm]
Irr_noise          = WALTZ
Irr_pwidth         = 50[us]
Relaxation_delay= 1[s]
Solvent            = DMSO-D6
Temp_get           = 23.5[dC]
Spin_get           = 15[Hz]
Probe_id           = 2564
```

AIMAN-EDA_PROTON.3
AIMAN-EDA

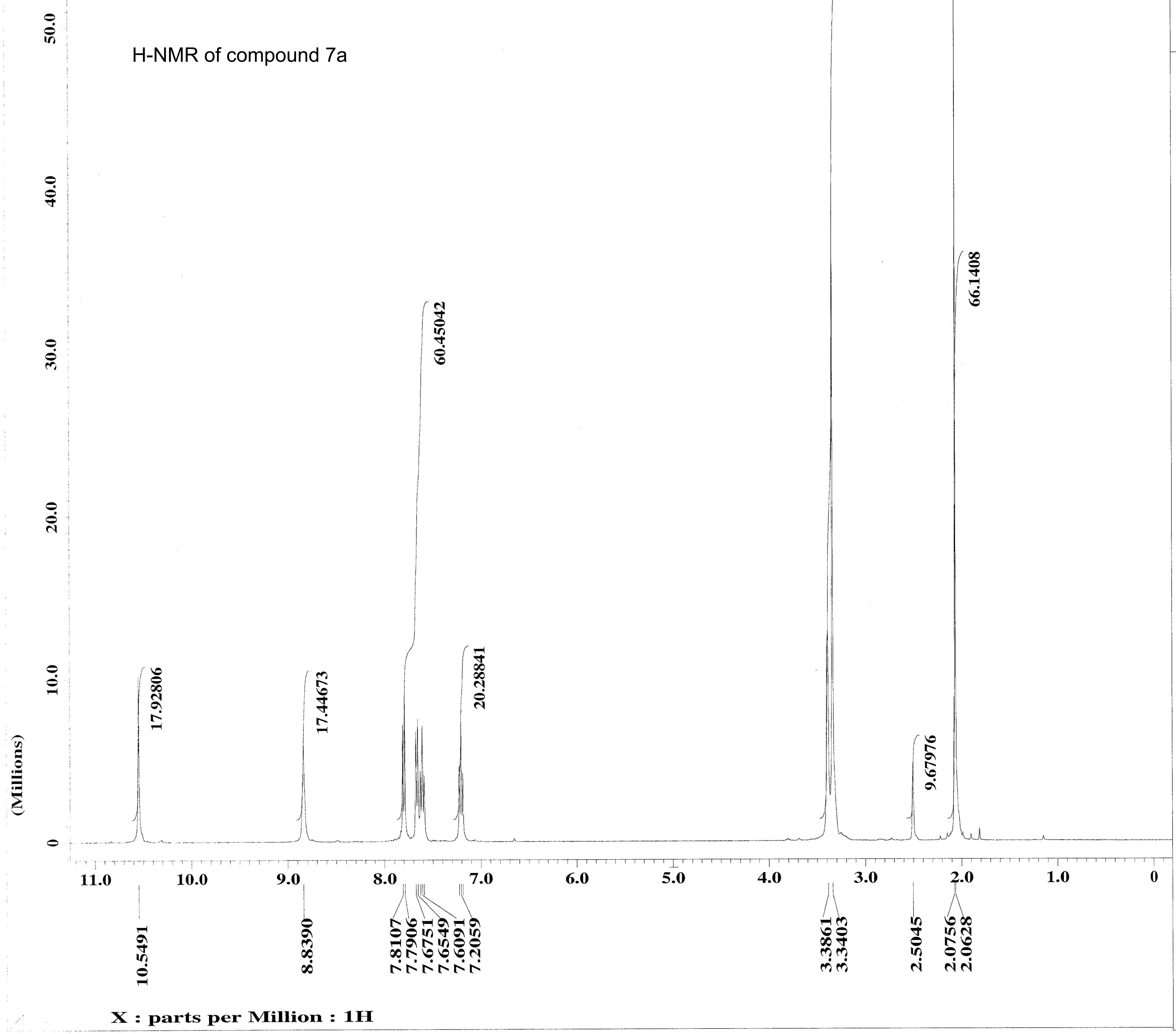


```
---- ACQUISITION PARAMETERS ----
File Name          = AIMAN-EDA_PROTON.3
Author             = DR. M. MARASHDAH
Sample ID          = AIMAN-EDA
Content            = AIMAN-EDA
Creation Date      = 17-APR-2013 09:21:07

Revision Date      = 17-APR-2013 12:28:23
Spec Site          = ECP400

Spec Type          = DELTA_NMR
Data Format        = 1D COMPLEX
Dimensions         = X
Dim Title          = 1H
Dim Size           = 16384
Dim Units          = [ppm]
Experiment         = single_pulse.exp
Field_strength     = 9.389766[T]
X_domain           = 1H
X_freq             = 399.7841973[MHz]
X_offset           = 5[ppm]
X_sweep            = 12.00480192[kHz]
X_points           = 16384
X_resolution       = 0.73275969[Hz]
Recvr_gain         = 19
Filter_mode        = BUTTERWORTH
X_prescans         = 0
Scans              = 8
Irr_noise          = WALTZ
Irr_pwidth         = 50[us]
Relaxation_delay= 4[s]
Solvent            = DMSO-D6
Temp_get           = 23.1[dC]
Spin_get           = 15[Hz]
Probe_id           = 2564
```

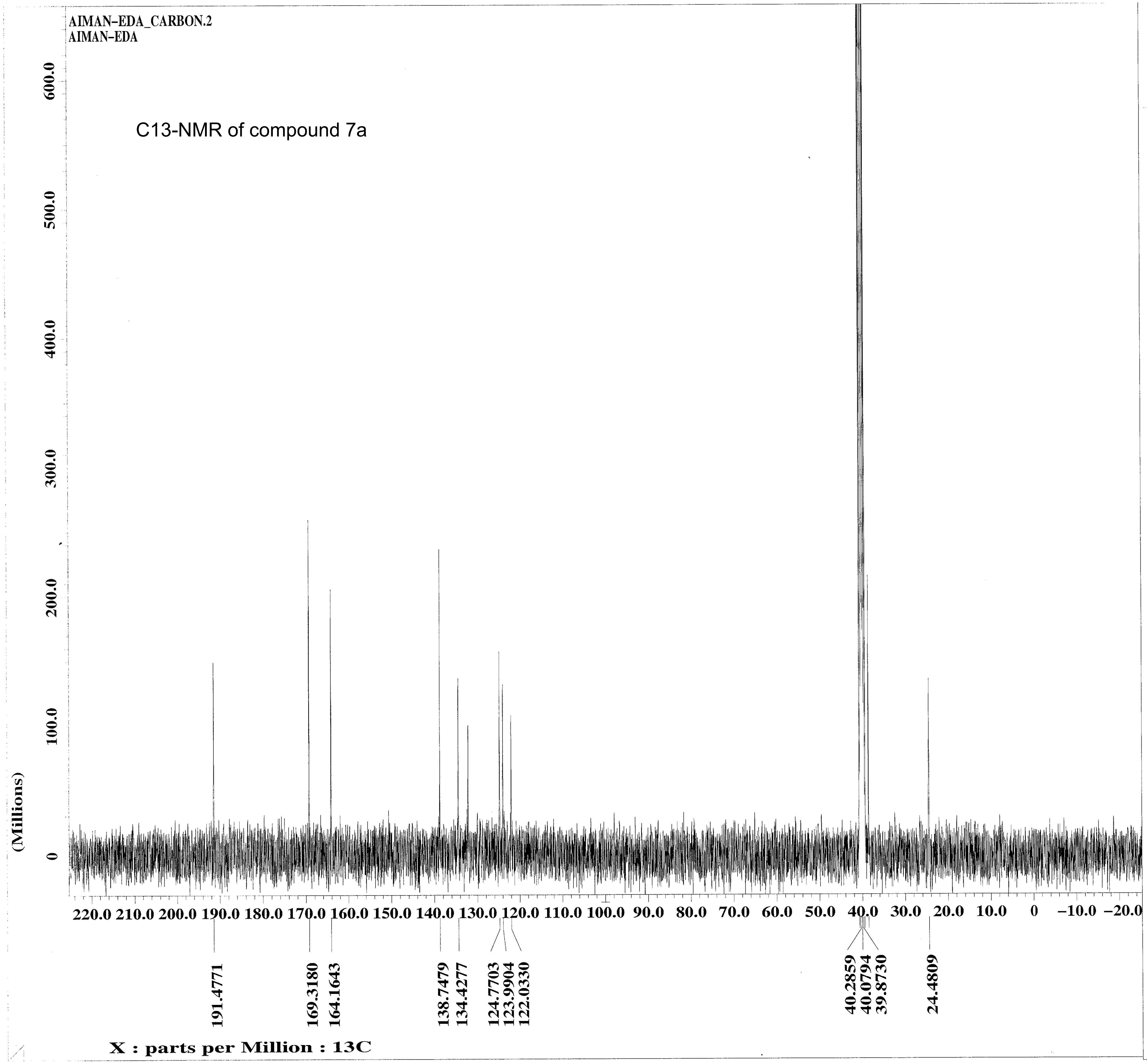

JEOL

---- ACQUISITION PARAMETERS ----

File Name	= AIMAN-EDA_CARBON.2
Author	= DR. M. MARASHDAH
Sample ID	= AIMAN-EDA
Content	= AIMAN-EDA
Creation Date	= 17-APR-2013 09:52:58
Revision Date	= 17-APR-2013 12:30:09
Spec Site	= ECP400
Spec Type	= DELTA_NMR
Data Format	= 1D COMPLEX
Dimensions	= X
Dim Title	= 13C
Dim Size	= 32768
Dim Units	= [ppm]
Experiment	= single_pulse_dec
Field_strength	= 9.389766[T]
X_domain	= 13C
X_freq	= 100.53535686[MHz]
X_offset	= 100[ppm]
X_sweep	= 25.18891688[kHz]
X_points	= 32768
X_resolution	= 0.7687282[Hz]
Recvr_gain	= 29
Filter_mode	= BUTTERWORTH
X_prescans	= 4
Scans	= 800
Irr_domain	= 1H
Irr_offset	= 5.0[ppm]
Irr_noise	= WALTZ
Irr_pwidth	= 50[us]
Relaxation_delay	= 1[s]
Solvent	= DMSO-D6
Temp_get	= 24[dC]
Spin_get	= 15[Hz]
Probe_id	= 2564

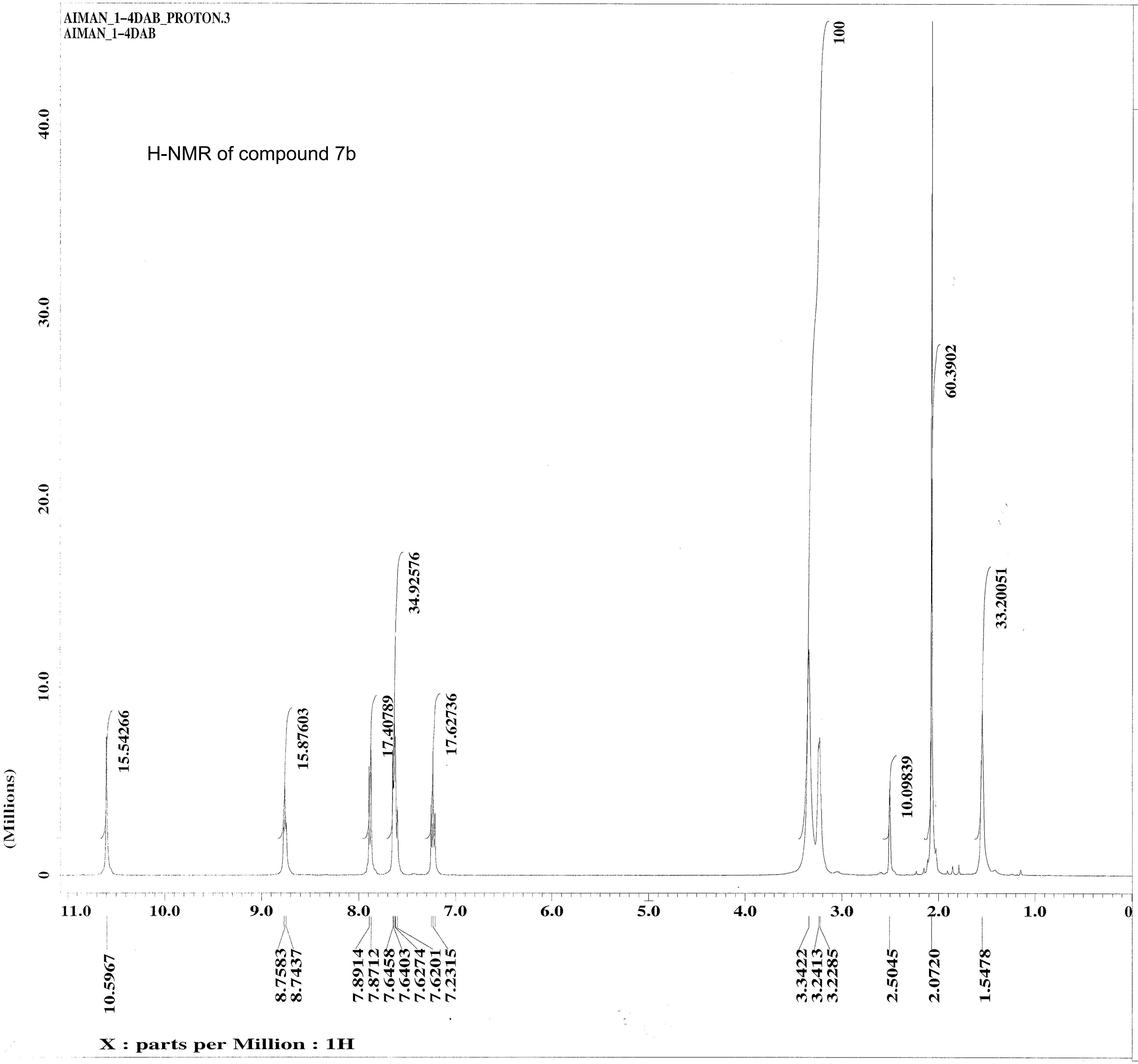

JEOL

---- ACQUISITION PARAMETERS ----

File Name	= AIMAN_1-4DAB_PROTON.3
Author	= DR. M. MARASHDAH
Sample ID	= AIMAN_1-4DAB
Content	= AIMAN_1-4DAB
Creation Date	= 22-APR-2013 10:58:32
Revision Date	= 22-APR-2013 13:33:34
Spec Site	= ECP400
Spec Type	= DELTA_NMR
Data Format	= 1D COMPLEX
Dimensions	= X
Dim Title	= 1H
Dim Size	= 16384
Dim Units	= [ppm]
Experiment	= single_pulse.exp
Field_strength	= 9.389766[T]
X_domain	= 1H
X_freq	= 399.7841973[MHz]
X_offset	= 5[ppm]
X_sweep	= 12.00480192[kHz]
X_points	= 16384
X_resolution	= 0.73275969[Hz]
Recvr_gain	= 17
Filter_mode	= BUTTERWORTH
X_prescans	= 0
Scans	= 8
Irr_noise	= WALTZ
Irr_pwidth	= 50[us]
Relaxation_delay	= 4[s]
Solvent	= DMSO-D6
Temp_get	= 23[dC]
Spin_get	= 16[Hz]
Probe_id	= 2564

AIMAN_1-4DAB_CARBON.2
AIMAN_1-4DAB

C13-NMR of compound 7b

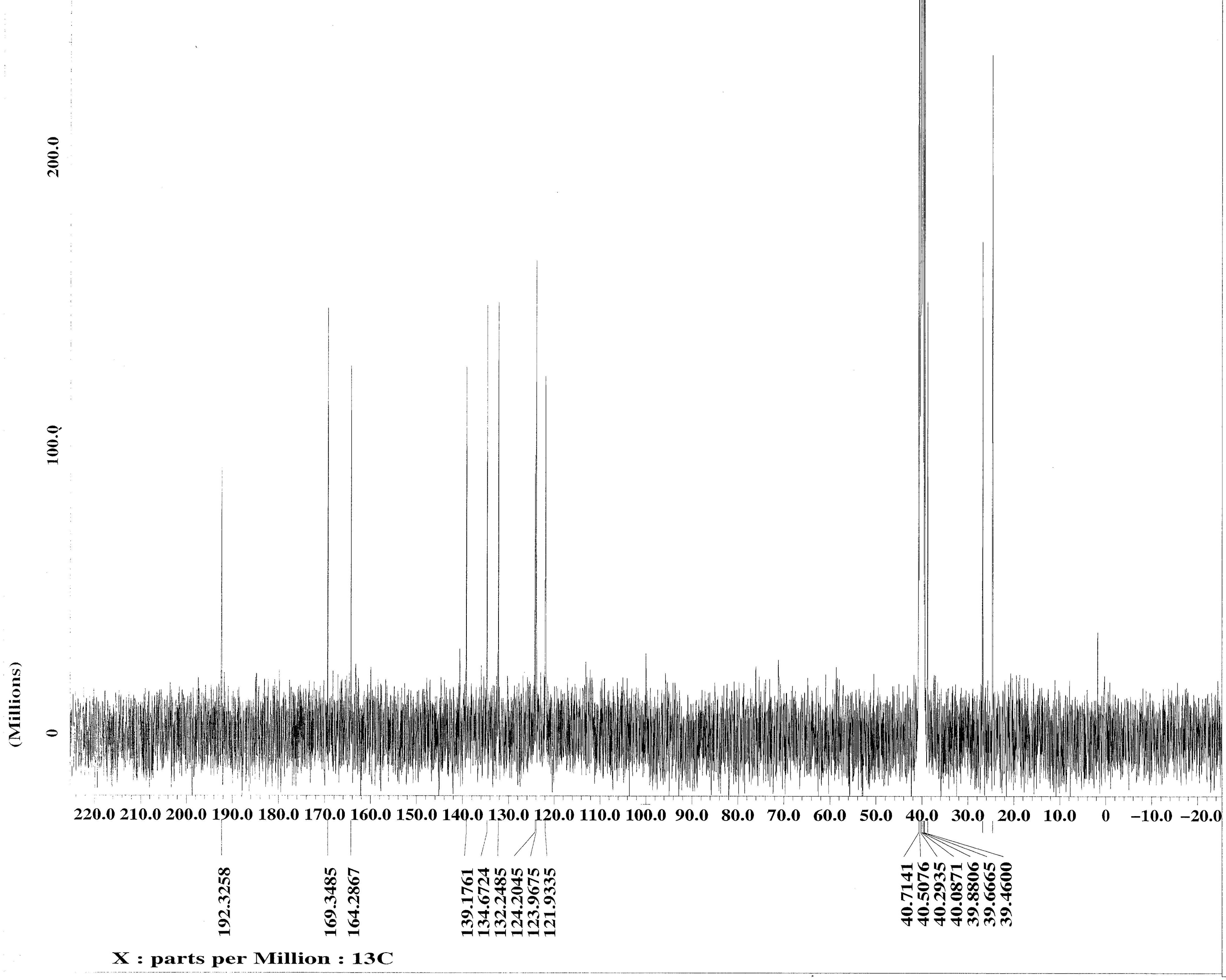

JEOL

```
---- ACQUISITION PARAMETERS ----
File Name          = AIMAN_1-4DAB_CARBON.2
Author             = DR. M. MARASHDAH
Sample ID          = AIMAN_1-4DAB
Content            = AIMAN_1-4DAB
Creation Date      = 22-APR-2013 11:38:52

Revision Date      = 22-APR-2013 13:34:47
Spec Site          = ECP400

Spec Type          = DELTA_NMR
Data Format        = 1D COMPLEX
Dimensions         = X
Dim Title          = 13C
Dim Size           = 32768
Dim Units          = [ppm]
Experiment         = single_pulse_dec
Field_strength     = 9.389766[T]
X_domain           = 13C
X_freq             = 100.53535686[MHz]
X_offset           = 100[ppm]
X_sweep            = 25.18891688[kHz]
X_points           = 32768
X_resolution       = 0.7687282[Hz]
Recvr_gain         = 27
Filter_mode        = BUTTERWORTH
X_prescans         = 4
Scans              = 1024
Irr_domain         = 1H
Irr_offset         = 5.0[ppm]
Irr_noise          = WALTZ
Irr_pwidth         = 50[us]
Relaxation_delay=  1[s]
Solvent            = DMSO-D6
Temp_get           = 24.2[dC]
Spin_get           = 16[Hz]
Probe_id           = 2564
```

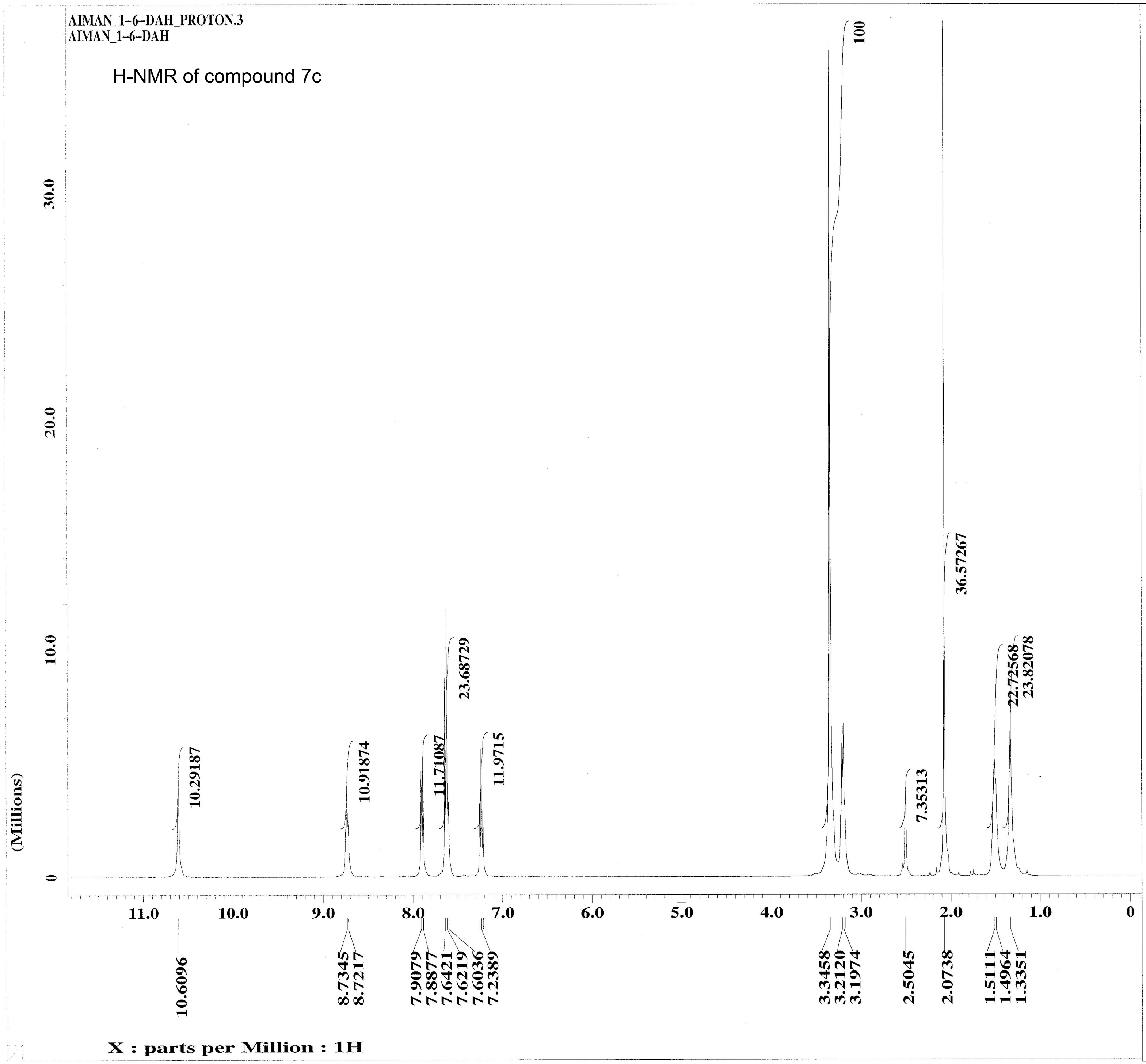


```
---- ACQUISITION PARAMETERS ----
File Name          = AIMAN_1-6-DAH_PROTON.3
Author             = DR. M. MARASHDAH
Sample ID          = AIMAN_1-6-DAH
Content            = AIMAN_1-6-DAH
Creation Date      = 28-APR-2013 12:55:28

Revision Date      = 28-APR-2013 14:31:10
Spec Site          = ECP400

Spec Type          = DELTA_NMR
Data Format        = 1D COMPLEX
Dimensions         = X
Dim Title          = 1H
Dim Size           = 16384
Dim Units          = [ppm]
Experiment         = single_pulse.exp
Field_strength     = 9.389766[T]
X_domain           = 1H
X_freq             = 399.7841973[MHz]
X_offset           = 5[ppm]
X_sweep            = 12.00480192[kHz]
X_points           = 16384
X_resolution       = 0.73275969[Hz]
Recvr_gain         = 17
Filter_mode        = BUTTERWORTH
X_prescans         = 0
Scans              = 8
Irr_noise          = WALTZ
Irr_pwidth         = 50[us]
Relaxation_delay= 4[s]
Solvent            = DMSO-D6
Temp_get           = 22.3[dC]
Spin_get           = 16[Hz]
Probe_id           = 2564
```
