# Supplementary material for: Microwave irradiation: synthesis and characterization of α-ketoamide and bis (α-ketoamide) derivatives via the ring opening of N-acetylisatin
Source: Chem Cent J. 2014 Apr 28;8:27. doi: 10.1186/1752-153X-8-27 (PMC4021159; doi:10.1186/1752-153X-8-27)
Supplement: Additional file 5 — 1H NMR spectra of compound of compound 8a. 13C NMR spectra of compound of compound 8a. 1H NMR spectra of compound of compound 8d. 13C NMR spectra of compound of compound 8d. [file 1752-153X-8-27-S5.pdf]

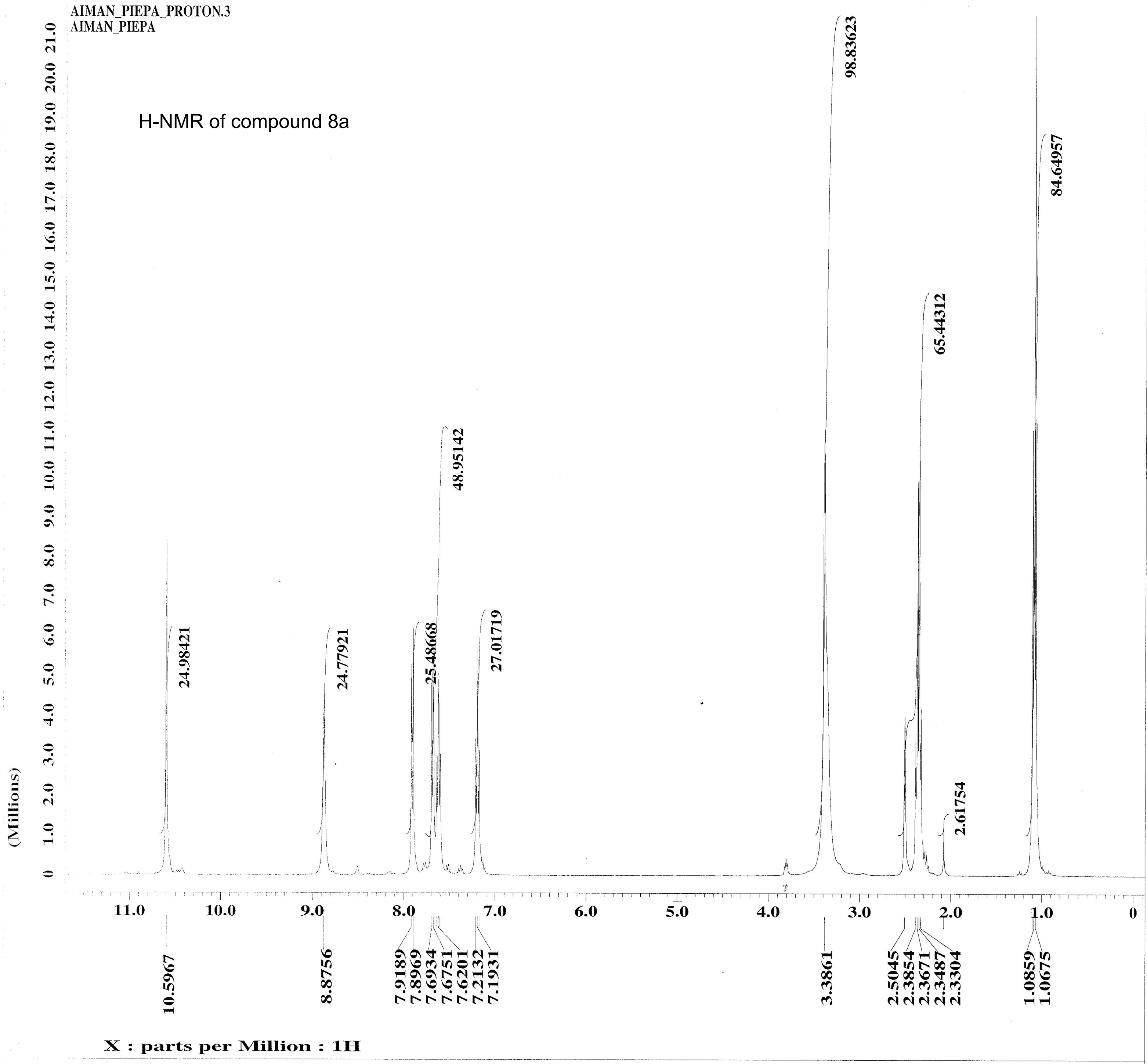

JEOL

```
---- ACQUISITION PARAMETERS ----
File Name          = AIMAN_PIEPA_PROTON.3
Author             = DR. M. MARASHDAH
Sample ID          = AIMAN_PIEPA
Content            = AIMAN_PIEPA
Creation Date      = 26-MAY-2013 10:16:56

Revision Date      = 26-MAY-2013 11:03:42
Spec Site          = ECP400

Spec Type          = DELTA_NMR
Data Format        = 1D COMPLEX
Dimensions         = X
Dim Title          = 1H
Dim Size           = 16384
Dim Units          = [ppm]
Experiment         = single_pulse.exp
Field_strength     = 9.389766[T]
X_domain           = 1H
X_freq             = 399.7841973[MHz]
X_offset           = 5[ppm]
X_sweep            = 12.00480192[kHz]
X_points           = 16384
X_resolution       = 0.73275969[Hz]
Recvr_gain         = 15
Filter_mode        = BUTTERWORTH
X_prescans         = 0
Scans              = 8
Irr_noise          = WALTZ
Irr_pwidth         = 50[us]
Relaxation_delay= 4[s]
Solvent            = DMSO-D6
Temp_get           = 21.8[dC]
Spin_get           = 15[Hz]
Probe_id           = 2564
```

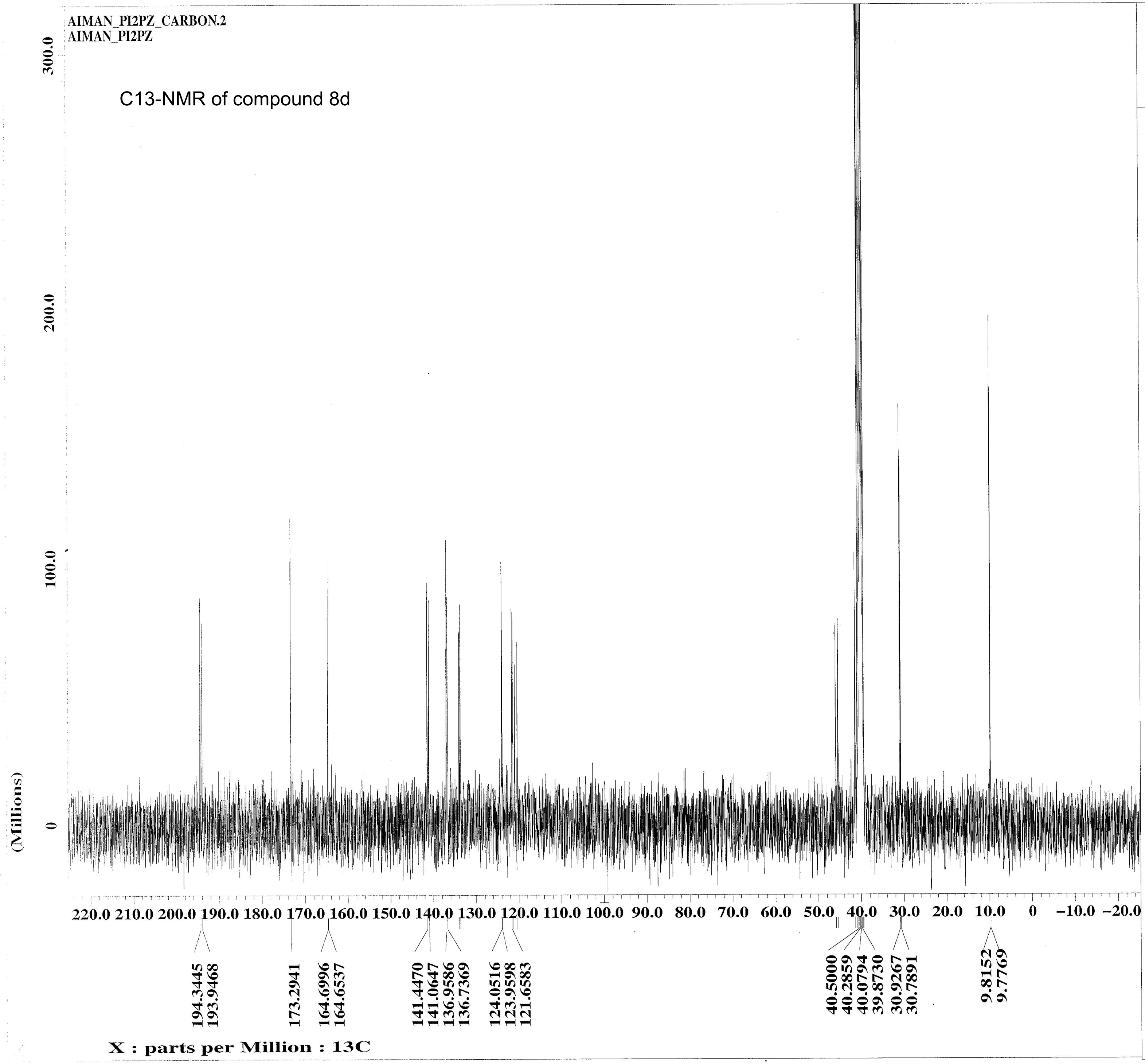

---- ACQUISITION PARAMETERS ----

File Name	= AIMAN_PI2PZ_CARBON.2
Author	= DR. M. MARASHDAH
Sample ID	= AIMAN_PI2PZ
Content	= AIMAN_PI2PZ
Creation Date	= 26-MAY-2013 11:45:03
Revision Date	= 26-MAY-2013 14:53:37
Spec Site	= ECP400
Spec Type	= DELTA_NMR
Data Format	= 1D COMPLEX
Dimensions	= X
Dim Title	= 13C
Dim Size	= 32768
Dim Units	= [ppm]
Experiment	= single_pulse_dec
Field_strength	= 9.389766[T]
X_domain	= 13C
X_freq	= 100.53535686[MHz]
X_offset	= 100[ppm]
X_sweep	= 25.18891688[kHz]
X_points	= 32768
X_resolution	= 0.7687282[Hz]
Recvr_gain	= 27
Filter_mode	= BUTTERWORTH
X_prescans	= 4
Scans	= 800
Irr_domain	= 1H
Irr_offset	= 5.0[ppm]
Irr_noise	= WALTZ
Irr_pwidth	= 50[us]
Relaxation_delay	= 1[s]
Solvent	= DMSO-D6
Temp_get	= 23.6[dC]
Spin_get	= 14[Hz]
Probe_id	= 2564

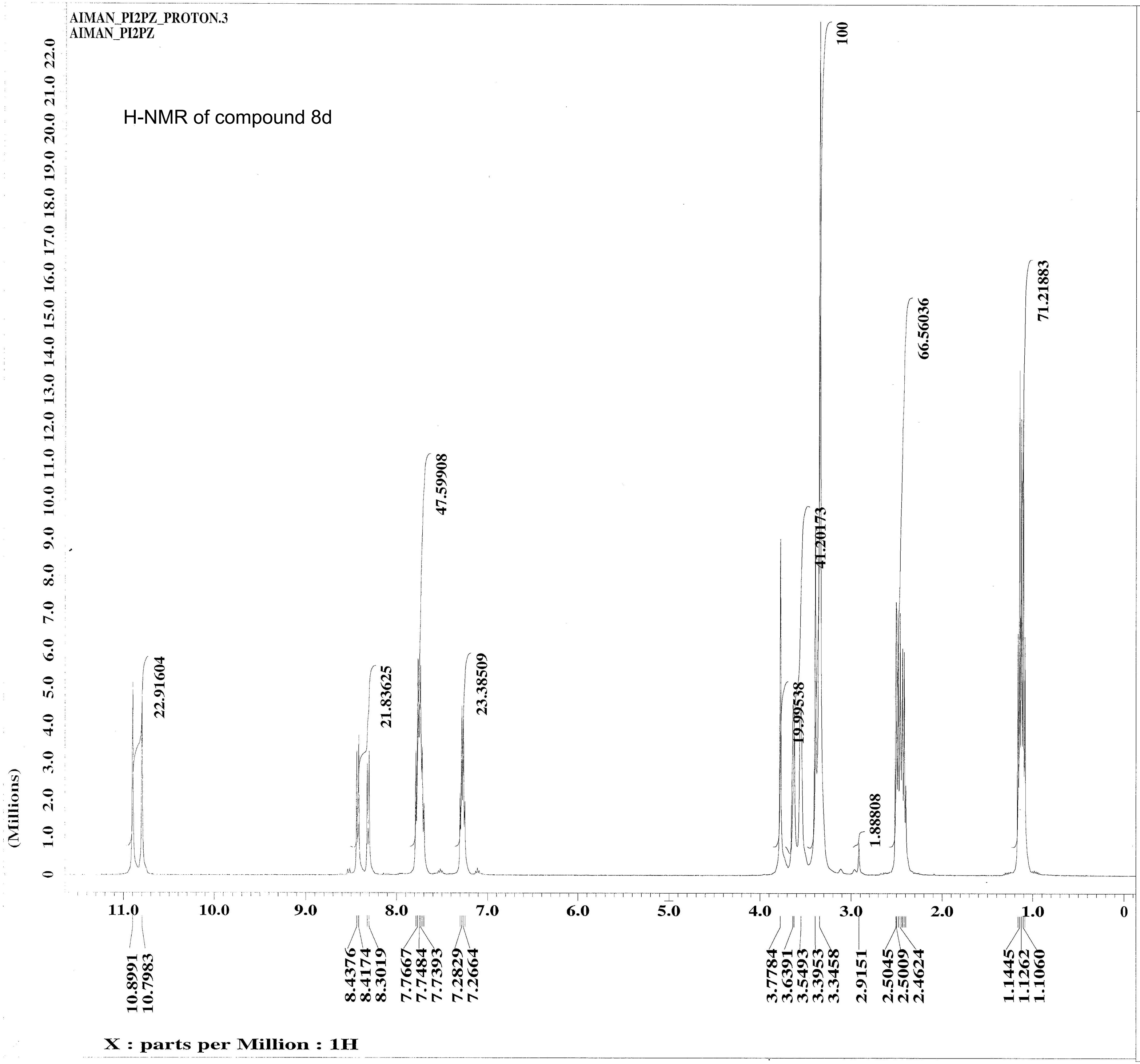

---- ACQUISITION PARAMETERS ----

```
File Name          = AIMAN_PI2PZ_PROTON.3
Author             = DR. M. MARASHDAH
Sample ID          = AIMAN_PI2PZ
Content            = AIMAN_PI2PZ
Creation Date      = 26-MAY-2013 11:13:19

Revision Date      = 26-MAY-2013 14:52:02
Spec Site          = ECP400

Spec Type          = DELTA_NMR
Data Format        = 1D COMPLEX
Dimensions         = X
Dim Title          = 1H
Dim Size           = 16384
Dim Units          = [ppm]
Experiment         = single_pulse.exp
Field_strength     = 9.389766[T]
X_domain           = 1H
X_freq             = 399.7841973[MHz]
X_offset           = 5[ppm]
X_sweep            = 12.00480192[kHz]
X_points           = 16384
X_resolution       = 0.73275969[Hz]
Recvr_gain         = 16
Filter_mode        = BUTTERWORTH
X_prescans         = 0
Scans              = 8
Irr_noise          = WALTZ
Irr_pwidth         = 50[us]
Relaxation_delay= 4[s]
Solvent            = DMSO-D6
Temp_get           = 22.4[dC]
Spin_get           = 14[Hz]
Probe_id           = 2564
```

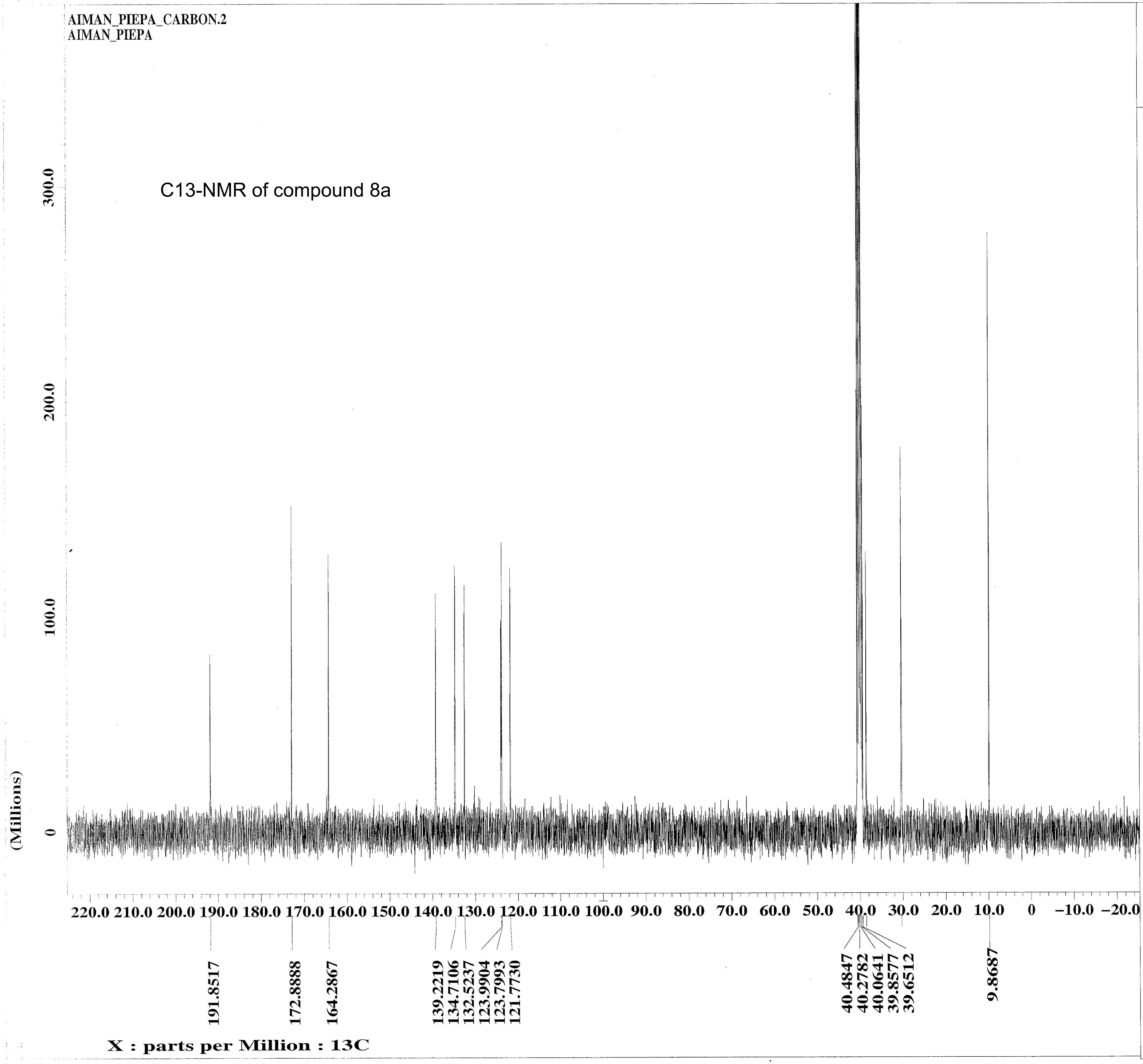

---- ACQUISITION PARAMETERS ----

File Name	= AIMAN_PIEPA_CARBON.2
Author	= DR. M. MARASHDAH
Sample ID	= AIMAN_PIEPA
Content	= AIMAN_PIEPA
Creation Date	= 26-MAY-2013 10:28:53
Revision Date	= 26-MAY-2013 11:06:12
Spec Site	= ECP400
Spec Type	= DELTA_NMR
Data Format	= 1D COMPLEX
Dimensions	= X
Dim Title	= 13C
Dim Size	= 32768
Dim Units	= [ppm]
Experiment	= single_pulse_dec
Field_strength	= 9.389766[T]
X_domain	= 13C
X_freq	= 100.53535686[MHz]
X_offset	= 100[ppm]
X_sweep	= 25.18891688[kHz]
X_points	= 32768
X_resolution	= 0.7687282[Hz]
Recvr_gain	= 28
Filter_mode	= BUTTERWORTH
X_prescans	= 4
Scans	= 280
Irr_domain	= 1H
Irr_offset	= 5.0[ppm]
Irr_noise	= WALTZ
Irr_pwidth	= 50[us]
Relaxation_delay	= 1[s]
Solvent	= DMSO-D6
Temp_get	= 22.8[dC]
Spin_get	= 14[Hz]
Probe_id	= 2564
