# Supplementary material for: Microwave irradiation: synthesis and characterization of α-ketoamide and bis (α-ketoamide) derivatives via the ring opening of N-acetylisatin
Source: Chem Cent J. 2014 Apr 28;8:27. doi: 10.1186/1752-153X-8-27 (PMC4021159; doi:10.1186/1752-153X-8-27)
Supplement: Additional file 6 — 1H NMR spectra of compound of compound 9a. 13C NMR spectra of compound of compound 9a. [file 1752-153X-8-27-S6.pdf]

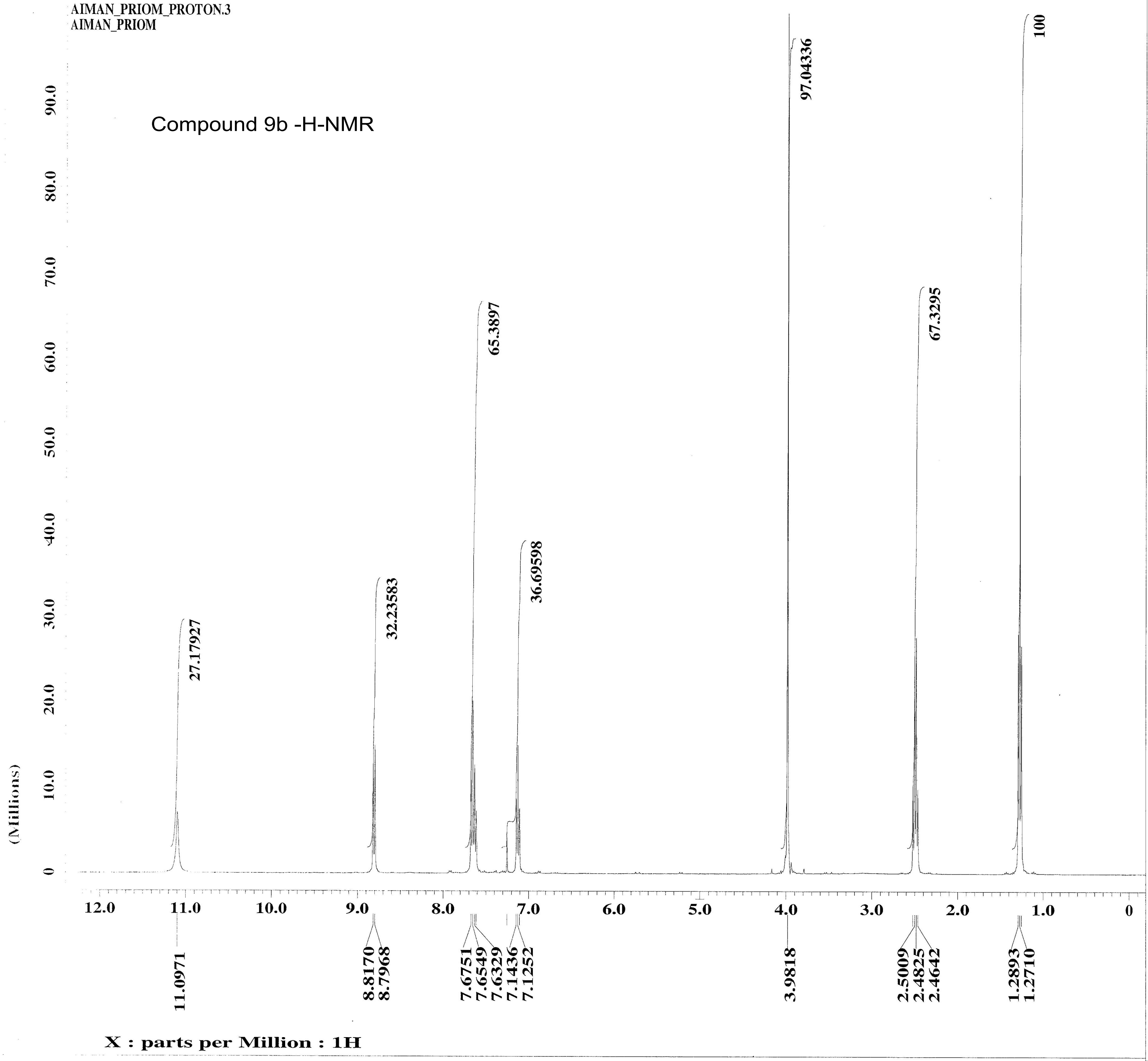

---- ACQUISITION PARAMETERS ----
File Name = AIMAN_PRIOM_PROTON.3
Author = DR. M. MARASHDAH
Sample ID = AIMAN_PRIOM
Content = AIMAN_PRIOM
Creation Date = 15-APR-2013 09:01:56

Revision Date = 15-APR-2013 12:05:24
Spec Site = ECP400

Spec Type = DELTA_NMR
Data Format = 1D COMPLEX
Dimensions = X
Dim Title = 1H
Dim Size = 16384
Dim Units = [ppm]
Experiment = single_pulse.exp
Field_strength = 9.389766[T]
X_domain = 1H
X_freq = 399.7841973[MHz]
X_offset = 5[ppm]
X_sweep = 12.00480192[kHz]
X_points = 16384
X_resolution = 0.73275969[Hz]
Recvr_gain = 17
Filter_mode = BUTTERWORTH
X_prescans = 0
Scans = 8
Irr_noise = WALTZ
Irr_pwidth = 50[us]
Relaxation_delay= 4[s]
Solvent = CHLOROFORM-D
Temp_get = 22.8[dC]
Spin_get = 14[Hz]
Probe_id = 2564

AIMAN_PRIOM_CARBON.2
AIMAN_PRIOM

Compound 9b -C-NMR

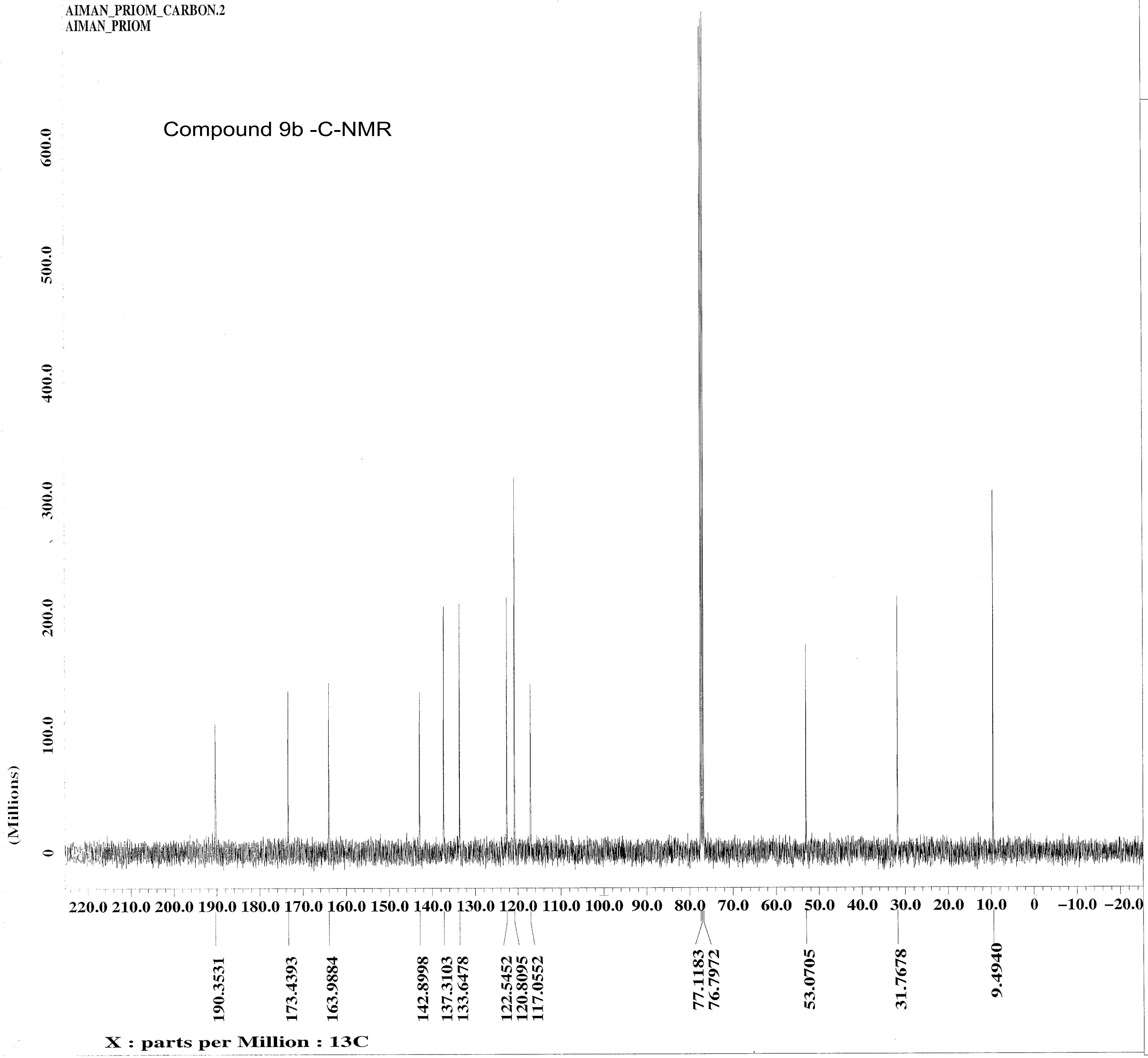

JEOL

```
---- ACQUISITION PARAMETERS ----
File Name          = AIMAN_PRIOM_CARBON.2
Author             = DR. M. MARASHDAH
Sample ID          = AIMAN_PRIOM
Content            = AIMAN_PRIOM
Creation Date      = 15-APR-2013 09:10:35

Revision Date      = 15-APR-2013 12:06:31
Spec Site          = ECP400

Spec Type          = DELTA_NMR
Data Format        = 1D COMPLEX
Dimensions         = X
Dim Title          = 13C
Dim Size           = 32768
Dim Units          = [ppm]
Experiment         = single_pulse_dec
Field_strength     = 9.389766[T]
X_domain           = 13C
X_freq             = 100.53535686[MHz]
X_offset           = 100[ppm]
X_sweep            = 25.18891688[kHz]
X_points           = 32768
X_resolution       = 0.7687282[Hz]
Recvr_gain         = 29
Filter_mode        = BUTTERWORTH
X_prescans         = 4
Scans              = 195
Irr_domain         = 1H
Irr_offset         = 5.0[ppm]
Irr_noise          = WALTZ
Irr_pwidth         = 50[us]
Relaxation_delay= 1[s]
Solvent            = CHLOROFORM-D
Temp_get           = 23.7[dC]
Spin_get           = 16[Hz]
Probe_id           = 2564
```
