# Supplementary material for: Microwave irradiation: synthesis and characterization of α-ketoamide and bis (α-ketoamide) derivatives via the ring opening of N-acetylisatin
Source: Chem Cent J. 2014 Apr 28;8:27. doi: 10.1186/1752-153X-8-27 (PMC4021159; doi:10.1186/1752-153X-8-27)
Supplement: Additional file 7 — 1H NMR spectra of compound of compound 9b. 13C NMR spectra of compound of compound 9b. [file 1752-153X-8-27-S7.pdf]

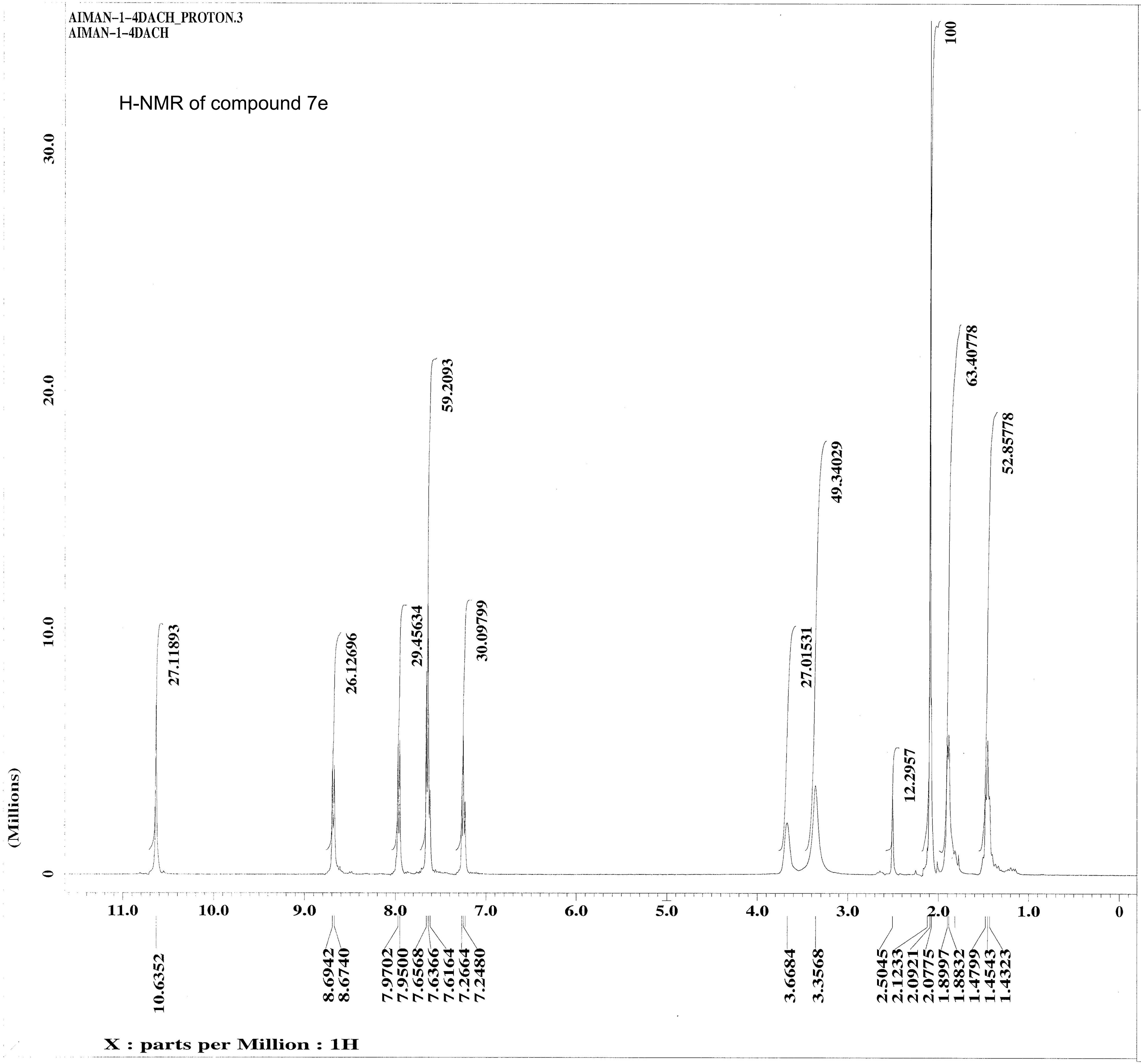

---- ACQUISITION PARAMETERS ----

```
File Name        = AIMAN-1-4DACH_PROTON.3
Author           = DR. M. MARASHDAH
Sample ID        = AIMAN-1-4DACH
Content          = AIMAN-1-4DACH
Creation Date    = 17-APR-2013 08:43:59

Revision Date    = 17-APR-2013 12:17:55
Spec Site        = ECP400

Spec Type        = DELTA_NMR
Data Format      = 1D COMPLEX
Dimensions       = X
Dim Title        = 1H
Dim Size         = 16384
Dim Units        = [ppm]
Experiment       = single_pulse.exp
Field_strength   = 9.389766[T]
X_domain         = 1H
X_freq           = 399.7841973[MHz]
X_offset         = 5[ppm]
X_sweep          = 12.00480192[kHz]
X_points         = 16384
X_resolution     = 0.73275969[Hz]
Recvr_gain       = 19
Filter_mode      = BUTTERWORTH
X_prescans       = 0
Scans            = 8
Irr_noise        = WALTZ
Irr_pwidth       = 50[us]
Relaxation_delay= 4[s]
Solvent          = DMSO-D6
Temp_get         = 22.8[dC]
Spin_get         = 14[Hz]
Probe_id         = 2564
```

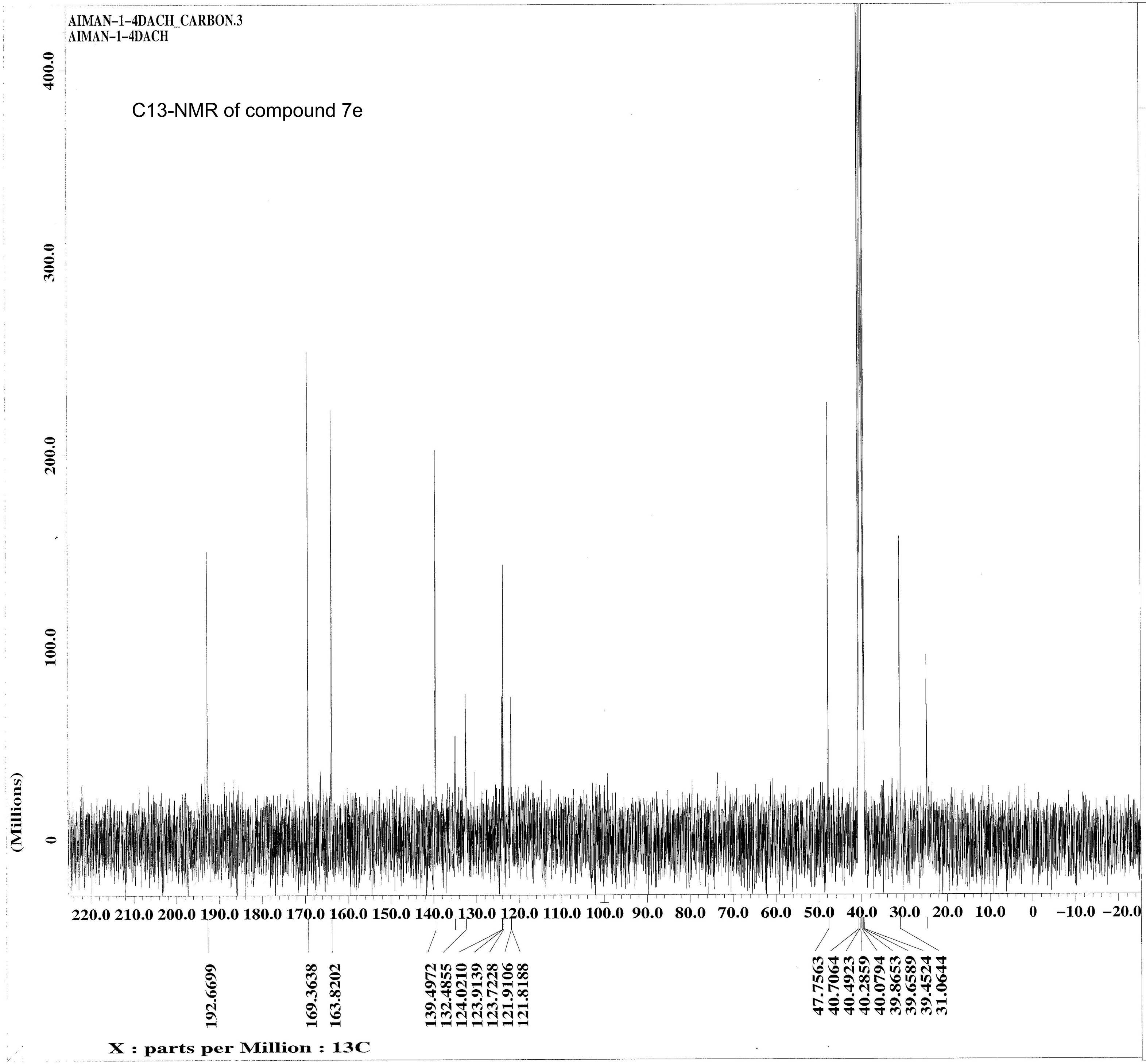


```
---- ACQUISITION PARAMETERS ----
File Name          = AIMAN-1-4DACH_CARBON.3
Author             = DR. M. MARASHDAH
Sample ID          = AIMAN-1-4DACH
Content            = AIMAN-1-4DACH
Creation Date      = 17-APR-2013 09:15:50

Revision Date      = 17-APR-2013 12:25:29
Spec Site          = ECP400

Spec Type          = DELTA_NMR
Data Format        = 1D COMPLEX
Dimensions         = X
Dim Title          = 13C
Dim Size           = 32768
Dim Units          = [ppm]
Experiment         = single_pulse_dec
Field_strength     = 9.389766[T]
X_domain           = 13C
X_freq             = 100.53535686[MHz]
X_offset           = 100[ppm]
X_sweep            = 25.18891688[kHz]
X_points           = 32768
X_resolution       = 0.7687282[Hz]
Recvr_gain         = 29
Filter_mode        = BUTTERWORTH
X_prescans         = 4
Scans              = 800
Irr_domain         = 1H
Irr_offset         = 5.0[ppm]
Irr_noise          = WALTZ
Irr_pwidth         = 50[us]
Relaxation_delay= 1[s]
Solvent            = DMSO-D6
Temp_get           = 23.7[dC]
Spin_get           = 14[Hz]
Probe_id           = 2564
```

H-NMR of compund 7d

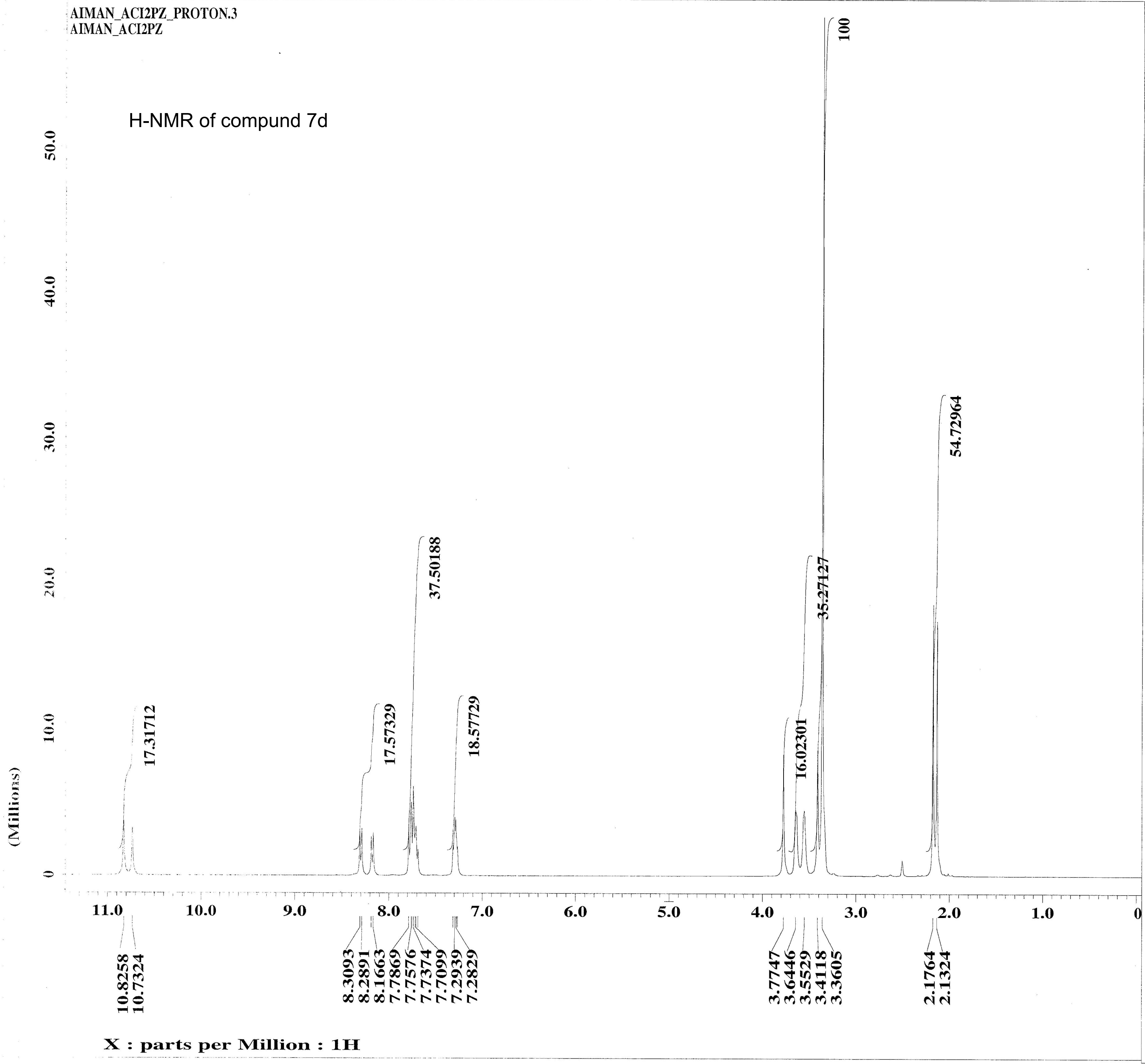

JEOL

```
---- ACQUISITION PARAMETERS ----
File Name         = AIMAN_ACI2PZ_PROTON.3
Author            = DR. M. MARASHDAH
Sample ID         = AIMAN_ACI2PZ
Content           = AIMAN_ACI2PZ
Creation Date     =  9-APR-2013 10:10:30

Revision Date     =  9-APR-2013 11:35:20
Spec Site         = ECP400

Spec Type         = DELTA_NMR
Data Format       = 1D COMPLEX
Dimensions        = X
Dim Title         = 1H
Dim Size          = 16384
Dim Units         = [ppm]
Experiment        = single_pulse.exp
Field_strength    = 9.389766[T]
X_domain          = 1H
X_freq            = 399.7841973[MHz]
X_offset          = 5[ppm]
X_sweep           = 12.00480192[kHz]
X_points          = 16384
X_resolution      = 0.73275969[Hz]
Recvr_gain        = 14
Filter_mode       = BUTTERWORTH
X_prescans        = 0
Scans             = 8
Irr_noise         = WALTZ
Irr_pwidth        = 50[us]
Relaxation_delay= 4[s]
Solvent           = DMSO-D6
Temp_get          = 22[dC]
Spin_get          = 14[Hz]
Probe_id          = 2564
```

AIMAN_ACI2PZ_CARBON.2
AIMAN_ACI2PZ

C13-NMR of compound 7d

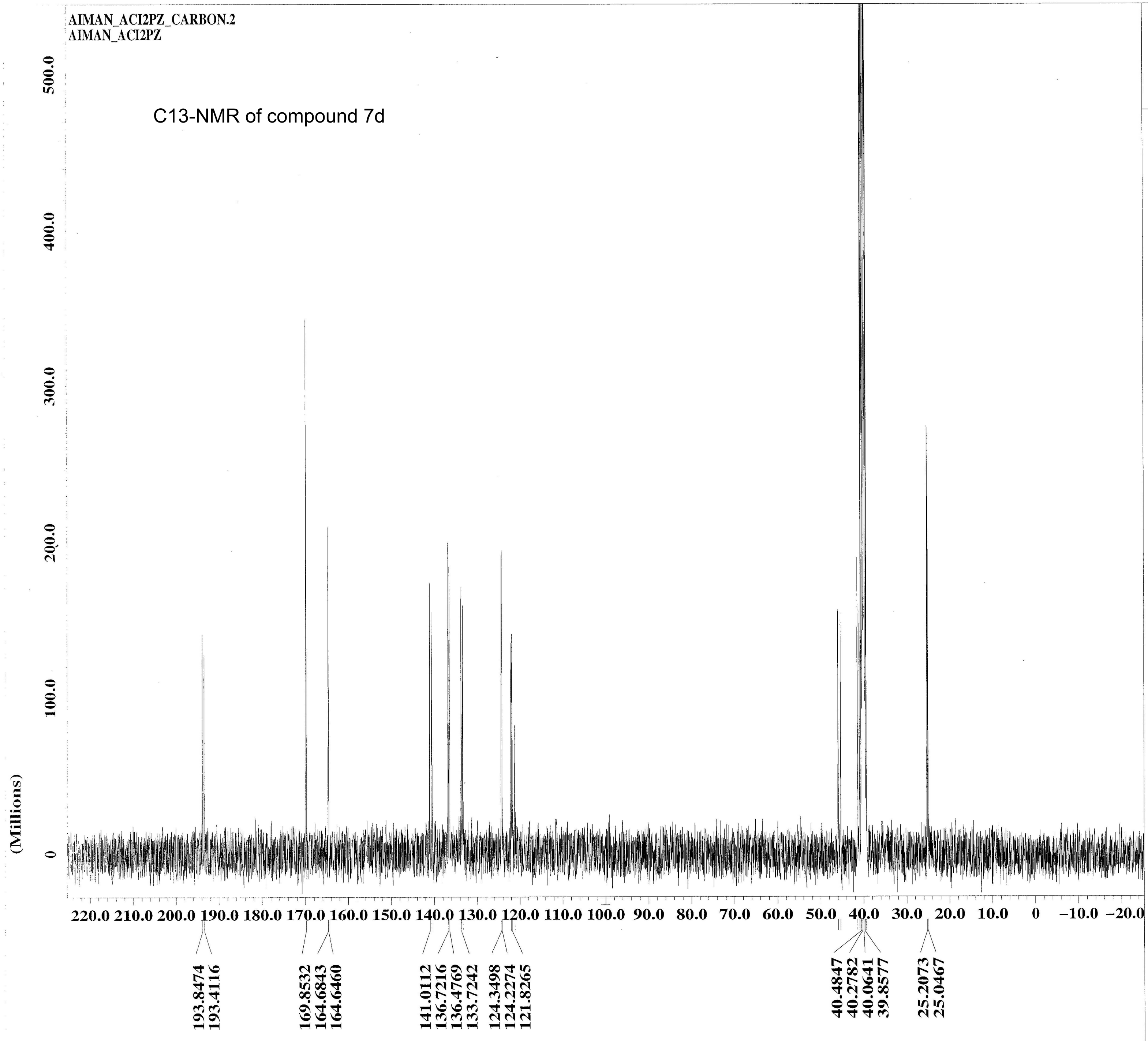

X : parts per Million : 13C

JEOL

```
---- ACQUISITION PARAMETERS ----
File Name          = AIMAN_ACI2PZ_CARBON.2
Author             = DR. M. MARASHDAH
Sample ID          = AIMAN_ACI2PZ
Content            = AIMAN_ACI2PZ
Creation Date      =  9-APR-2013 10:45:30

Revision Date      =  9-APR-2013 11:36:42
Spec Site          = ECP400

Spec Type          = DELTA_NMR
Data Format        = 1D COMPLEX
Dimensions         = X
Dim Title          = 13C
Dim Size           = 32768
Dim Units          = [ppm]
Experiment         = single_pulse_dec
Field_strength     = 9.389766[T]
X_domain           = 13C
X_freq             = 100.53535686[MHz]
X_offset           = 100[ppm]
X_sweep            = 25.18891688[kHz]
X_points           = 32768
X_resolution       = 0.7687282[Hz]
Recvr_gain         = 27
Filter_mode        = BUTTERWORTH
X_prescans         = 4
Scans              = 884
Irr_domain         = 1H
Irr_offset         = 5.0[ppm]
Irr_noise          = WALTZ
Irr_pwidth         = 50[us]
Relaxation_delay= 1[s]
Solvent            = DMSO-D6
Temp_get           = 23.2[dC]
Spin_get           = 15[Hz]
Probe_id           = 2564
```
