# Supplementary material for: Microwave irradiation: synthesis and characterization of α-ketoamide and bis (α-ketoamide) derivatives via the ring opening of N-acetylisatin
Source: Chem Cent J. 2014 Apr 28;8:27. doi: 10.1186/1752-153X-8-27 (PMC4021159; doi:10.1186/1752-153X-8-27)
Supplement: Additional file 3 — 1H NMR spectra of compound of compound 7a. 13C NMR spectra of compound of compound 7a. 1H NMR spectra of compound of compound 7b. 13C NMR spectra of compound of compound 7b. [file 1752-153X-8-27-S3.pdf]

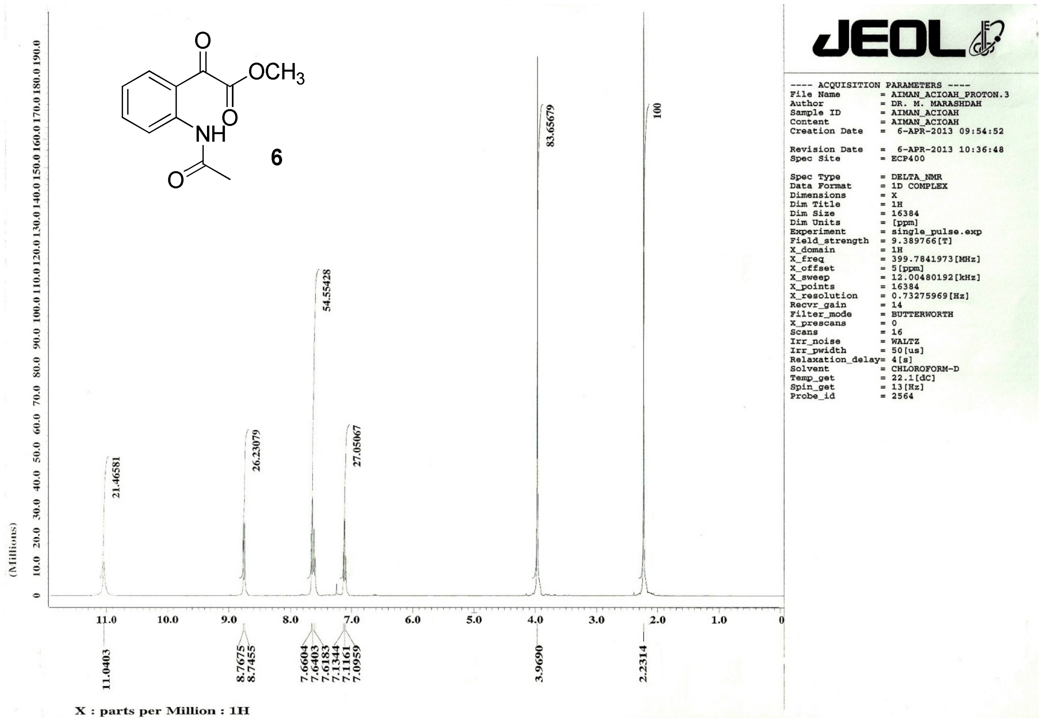

OCH3
NH
6
JEOL
---- ACQUISITION PARAMETERS ----
File Name = AIMAN_ACIOAH_PROTON.3
Author = DR. M. MARASHDAH
Sample ID = AIMAN_ACIOAH
Content = AIMAN_ACIOAH
Creation Date = 6-APR-2013 09:54:52
Revision Date = 6-APR-2013 10:36:48
Spec Site = ECP400
Spec Type = DELTA_NMR
Data Format = 1D COMPLEX
Dimensions = X
Dim Title = 1H
Dim Size = 16384
Dim Units = [ppm]
Experiment = single_pulse.exp
Field_strength = 9.389766[T]
X_domain = 1H
X_freq = 399.7841973[MHz]
X_offset = 5[ppm]
X_sweep = 12.00480192[kHz]
X_points = 16384
X_resolution = 0.73275969[Hz]
Recvr_gain = 14
Filter_mode = BUTTERWORTH
X_prescans = 0
Scans = 16
Irr_noise = WALTZ
Irr_pwidth = 50[us]
Relaxation_delay= 4[s]
Solvent = CHLOROFORM-D
Temp_get = 22.1[dC]
Spin_get = 13[Hz]
Probe_id = 2564
(Millions)
21.46581
26.23079
54.55428
27.05067
83.65679
100
11.0403
8.7675
8.7455
7.6604
7.6403
7.6183
7.1344
7.1161
7.0959
3.9690
2.2314
X : parts per Million : 1H

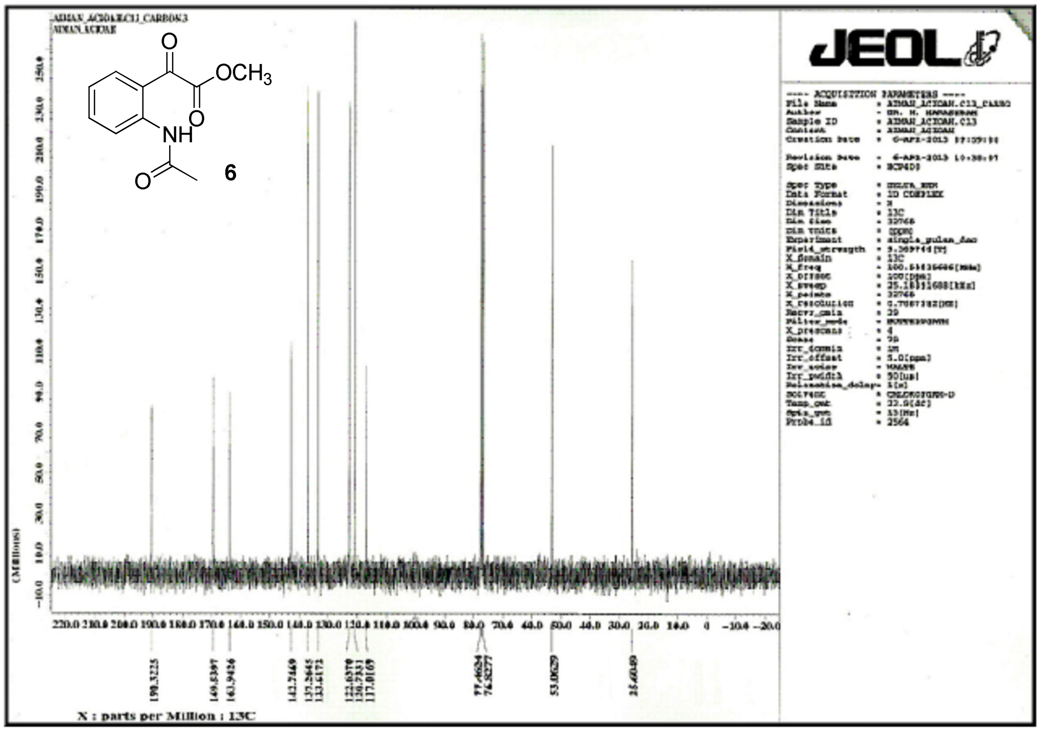

AIMAN_ACIOAH.C13_CARBON3
OCH3
NH
6
JEOL
---- ACQUISITION PARAMETERS ----
X : parts per Million : 13C
